# Role of Heme Oxygenase as a Modulator of Heme-Mediated Pathways

**DOI:** 10.3390/antiox8100475

**Published:** 2019-10-11

**Authors:** J. Catharina Duvigneau, Harald Esterbauer, Andrey V. Kozlov

**Affiliations:** 1 Institute for Medical Biochemistry, University of Veterinary Medicine, Veterinaerplatz 1, 1210 Vienna, Austria; catharina.duvigneau@vetmeduni.ac.at; 2 Department of Laboratory Medicine, Medical University of Vienna, 1210 Vienna, Austria; harald.esterbauer@meduniwien.ac.at; 3 Ludwig Boltzmann Institute for Experimental and Clinical Traumatology, 1200 Vienna, Austria; 4 Laboratory of Navigational Redox Lipidomics, Department of Human Pathology, IM Sechenov Moscow State Medical University, 119992 Moscow, Russia

**Keywords:** heme oxygenase, free heme, free iron, intracellular signaling, oxidative stress, biliverdin, carbon monoxide

## Abstract

The heme oxygenase (HO) system is essential for heme and iron homeostasis and necessary for adaptation to cell stress. HO degrades heme to biliverdin (BV), carbon monoxide (CO) and ferrous iron. Although mostly beneficial, the HO reaction can also produce deleterious effects, predominantly attributed to excessive product formation. Underrated so far is, however, that HO may exert effects additionally via modulation of the cellular heme levels. Heme, besides being an often-quoted generator of oxidative stress, plays also an important role as a signaling molecule. Heme controls the anti-oxidative defense, circadian rhythms, activity of ion channels, glucose utilization, erythropoiesis, and macrophage function. This broad spectrum of effects depends on its interaction with proteins ranging from transcription factors to enzymes. In degrading heme, HO has the potential to exert effects also via modulation of heme-mediated pathways. In this review, we will discuss the multitude of pathways regulated by heme to enlarge the view on HO and its role in cell physiology. We will further highlight the contribution of HO to pathophysiology, which results from a dysregulated balance between heme and the degradation products formed by HO.

## 1. Introduction

The last decades have brought to light an exciting spectrum of pathways that are maintained and modulated by heme oxygenase (HO). HO is the rate-limiting enzyme in the degradation of heme, resulting in the formation of equivalent amounts of biliverdin (BV), carbon monoxide (CO) and ferrous iron (Fe^2+^). In mammalian tissues, BV is rapidly reduced to bilirubin (BR) by the cytosolic enzyme biliverdin reductase (BVR). The HO system is not only an essential component of the heme and iron homeostasis but also indispensable for providing adaptation to cell stress. 

For most of the HO-mediated effects, two principal processes account, (i) the degradation of heme, and (ii) the generation of its reaction products. Two functionally active isoforms of HO have been described, the inducible and highly dynamic HO-1, and the constitutive HO-2, which is much less regulated [1]. They differ in respect to their tissue distribution and function. A third isoform (HO-3) is nearly identical to HO-2 (90%) in its amino acid sequence, but shows only low enzyme activity [2].

Most attention has been paid to HO-1, which is a member of the heat shock protein family (syn. HSP32) and an important component for the cellular stress control. In contrast to HO-2 knock-out mice, which show only minor physiological derangements and remain fertile [3], HO-1 knock-out mice display a high mortality in-utero and suffer from severe chronic inflammation. Further, adult HO-1-deficient animals develop both, serum iron deficiency and pathological iron overload, indicating that HO-1 is required for iron mobilization and distribution [4,5]. The only known HO-1 deficiency in a human patient showed multiple inflammatory complications and an early death [6], indicating the central role of HO-1 in normal mammalian development [4,7]. HO-2 has been less studied, possibly because it is barely regulated at its expression level. However, it contributes nearly completely to the HO activity in testis, endothelial cells, and particularly in the brain [8], underpinning the relevance of HO-2 for the proper function of these tissues [9,10]. Findings suggest that HO may also exert some of its modulatory effects independently of the catalytic activity. However, HO is mostly known for the impressive multitude of physiologic and pathophysiologic pathways regulated by virtue of its reaction products.

The unique HO products CO and BV/BR contribute to tissue function and protection by multiple mechanisms. The role of HO and its products supporting cytoprotection has been thoroughly addressed and discussed in several recent reviews [11,12,13,14,15]. Therefore, we will focus on properties of HO arising from aberrant HO activity that are less considered.

One consequence of an accelerated HO reaction is the accumulation of reaction products to critical levels. This applies to iron, which can cause severe counterproductive effects, if insufficiently sequestered. Other consequences of aberrant biological effects of HO are related to the role of heme. Heme is a pro-oxidative molecule when excessive, but it is also required at sufficient levels to fulfil important signaling functions. The intracellular heme pool is dynamic and the result of different fluxes. Import and export processes, biochemical synthesis, trafficking between organelles, and the assembly into hemoproteins all affect heme levels. The enzymatic degradation of heme will modify these fluxes and HO may therefore act as a regulator of heme-mediated signaling.

In this review we will summarize the pivotal role of HO for amelioration vs aggravation of diseases and recapitulate known information regarding the effects of decreased heme availability and increased product generation accounted by a sustained HO activity, including the newly discovered form of iron-mediated cell death, ferroptosis [16,17,18].

## 2. Pivotal Role of Heme Oxygenase Reaction—Salutary vs. Deleterious Effects

### 2.1. Beneficial Role of HO

Under physiological conditions, most tissues express only low levels of HO-1. Exceptions are the cells of the reticuloendothelial system, which are challenged with high levels of heme, due to the removal of aged erythrocytes. However, besides its own substrate, nearly all stressful conditions, such as hypoxia, ischemia/reperfusion, heavy metal exposure, fungal toxins, inflammatory agents, ultraviolet irradiation rapidly induce HO-1 [12,13,14,19,20,21,22,23]. Overwhelming data clearly show that high enzymatic activity of HO is a prerequisite for the well-recognized cytoprotective effects. Classically speaking, HO exerts anti-oxidative and cytoprotective effects by two synergistically operating measures. The first is the removal of heme, which can catalyze the formation of damaging reactive oxygen species (ROS), when insufficiently chaperoned as outlined in details in Section 2.3. Therefore, HO-1 induction significantly contributes to protection of the endothelium under conditions of elevated hemolysis [24]. The second measure is the increased generation of HO reaction products with distinct cytoprotective properties [25], which we will summarize in the following part.

BV/BR is considered as an important endogenous antioxidant system [26], although it has been shown that BVR-mediated redox cycle of BR/BV may have a less important role as a cellular antioxidant defense mechanism than generally assumed [27]. However, many studies have reported cytoprotective effects of BR that are based on its immunomodulatory capabilities [28,29,30,31,32,33].

**Figure 1:**
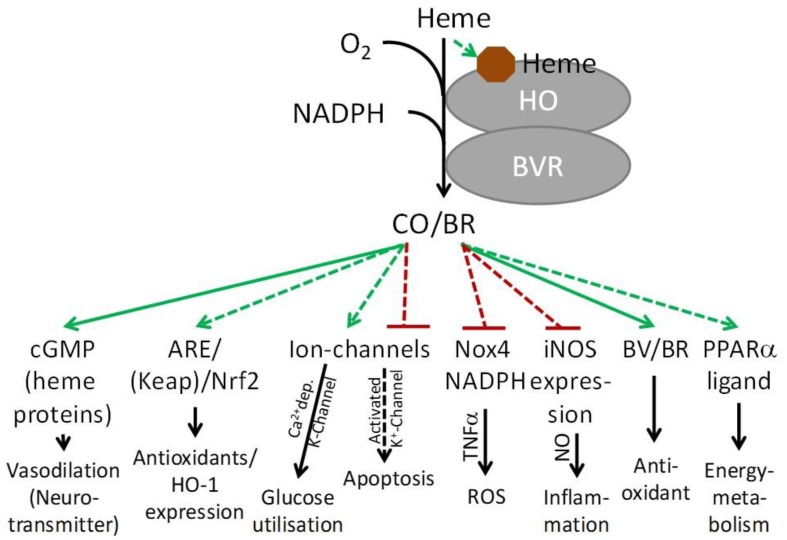
Overview of the predominant mechanisms underlying HO product-mediated cell protection. The two products of HO activity, namely, CO and BV/BR, are associated with the beneficial role of this enzyme. In stimulating cGMP synthesis, CO acts similarly to another gas messenger, NO. It further activates intracellular signaling cascades, involving Nrf2, which provides an anti-oxidative defense, and by interacting with ion channels for modulating glucose metabolism and inhibiting apoptosis. An anti-inflammatory action of BV/BR is predominantly executed by inhibiting generation of reactive oxygen and nitrogen species, via NOX and iNOS. Further, BR/BV possesses potent antioxidant capacity towards oxidation of biomolecules. Recently, BR acts as ligand of PPARα, thereby controlling lipid and energy homeostasis. Solid arrows indicate the most considered protective pathways. Abbreviations: ARE, antioxidant response elements; cGMP, cyclic guanosine monophosphate; iNOS, inducible NO-synthase; Keap, Kelch-like ECH-associated protein 1; Nrf2, nuclear-factor-E2-related factor-2; NO, nitric oxide; Nox4, NADPH oxidase (isoform 4); ROS, reactive oxygen species. PPAR, peroxisome proliferator activated receptor.

Besides these well-accepted roles, BR has recently been shown to act as a transcriptional regulator via peroxisome proliferator-activated receptor (PPAR)α [34]. Upon activation by BR, PPARα was shown to suppress lipid accumulation [35,36]. PPARα belongs to the PPAR family of lipid sensors, which are ligand activated transcription factors that modulate gene expression in a hormone-like fashion. Activation of PPARα affects energy storage and expenditure, as well as lipid and fatty acid homeostasis [37,38]. Loss of BVR induces alterations of insulin signaling, lipid accumulation, and hepatic steatosis, suggesting an important role for BR preventing lipotoxicity [39,40,41]. Apart from many other important functions, BVR also regulates glucose metabolism (for review see [42]). This entirely unexpected role of BR adds a new facet to this interesting part of the heme degradation pathway and dramatically changes the perception of BR from a metabolic waste to a metabolic signaling molecule (see Figure 1).

CO is a gaseous mediator with multiple properties, such as vasodilatory, anti-inflammatory, anti-proliferative, and anti-apoptotic activities (see Figure 1). In neuronal tissues CO plays a particular role, since it has been shown to act as neurotransmitter [43], regulator of ion channels [44] and to be required for memory consolidation [45]. The role of endogenous CO as a signaling molecule, particularly its role as activator of guanylate cyclase, has been excellently reviewed recently [46], and will therefore not be discussed in detail. 

During the last decades, the cytoprotective properties of the HO reaction have been extensively elucidated and are reviewed in different contexts in several outstanding papers (see [1,10,23,47,48,49,50,51]). However, in spite of their often-quoted beneficial aspects, an enhanced product formation due to excessive upregulation/activity of HO can also produce adverse effects. This aspect will be outlined in the next chapter. 

### 2.2. Absense of Cytoprotection or Aggravation of Disease by Upregulated HO

Various solid and blood cancers exploit HO to modulate pathways facilitating proliferation, progression of disease and chemoresistance (for review see: [52,53]). Further, HO-1 also supports cancer progression through modulating tumor microenvironment and expression of angiogenic factors [54,55,56]. Chemo-, radio- and photodynamic therapy appear to select for HO-1 overexpressing tumor cells, which are tolerant to the adverse stress conditions associated with treatment. Recently it has been shown that HO-1 overexpression and nuclear translocation, a mechanism occurring preferentially under pathological conditions, is associated with transcription regulating roles of HO-1, which are independent of the generation of HO products [57]. Together with the anti-oxidative and anti-apoptotic action of the HO products BR and CO exploited by tumor cells, these findings suggest HO-1 inhibition as a suitable therapy against different cancer types [55], such as melanoma [58], prostate cancer cells [59], as well as various leukemia malignancies [60,61]. In contrast to normal cells, many tumor cells are particularly vulnerable to inhibition of HO, because they possess reduced levels of other anti-oxidative enzyme systems, such as catalase, superoxide dismutase and glutathione peroxidase [62,63,64,65]. The promising results obtained in different experimental tumor systems using HO inhibitors or silencing of HO-1 [66,67,68] have encouraged the development of novel anti-cancer therapeutics [69,70,71,72]. These new types of HO inhibitors are water soluble and do not interfere with other heme containing enzymes, such as cytochrome P450 or iNOS [72], which has been described for higher concentrations of the classical metalloporphyrin-HO inhibitors [73]. These novel inhibitors are better suited for systemic application, and possibly serve not only as future anticancer drug, but also for the treatment of other diseases that are associated with an unfavorable upregulation of HO.

However, some cell types, including certain cancer cells, display an increased susceptibility to chemically induced cell death, despite upregulation of HO. It has been shown that chronic HO-1 upregulation sensitizes cells to electrophilic agents and ROS mediated injury [74,75,76,77]. Upon chronic HO-1 overexpression, adverse effects probably elicited by excessive product formation prevail (for review see: [78,79]). It has been shown that a several-fold induction of HO-1 counteracted the cytoprotective effects of its basal expression [80]. In neuronal tissue, HO-1 overexpression was frequently not associated with cytoprotection, but with an enhanced neuronal injury [77,81,82]. Also, low-grade inflammation may be caused by sustained upregulation of HO, which may be associated with polymorphism of the HO-1 promotor region [83]. This may explain the controversy of reports regarding the role of HO in onset and progression of metabolic syndrome and diabetes [21,84]. However, the thorough analysis of studies conducted in human population and a comprehensive mechanistic approach suggest that HO is causally involved in the onset and development of metabolic disease [21]. It has been further shown that HO-1 promotes delayed wound healing in diabetes [85], and is also associated with the progression of diabetic nephropathy [86].

All these findings demonstrate the diagnostic value of the knowledge of the displayed tissue HO-activity in various chronic diseases. Analysis of HO-1 expression in cancer biopsies is already used as a diagnostic tool and for the follow-up of therapeutic approaches [87,88,89,90,91]. Monitoring HO expression or activity will optimize treatment of other diseases and may help individual tailoring of therapeutic interventions.

### 2.3. Mechanisms Underlying Deleterious Effects of HO

Some of the deleterious effects of HO are attributed to the excessive formation of its products, particularly the accumulation of free ferrous iron. Free intracellular iron demands a sufficient cellular iron chelating capacity to cope with its pro-oxidative character. Additionally, an increased HO activity will lead to an increased consumption of NADPH. Cytosolic NADPH plays a role in numerous cellular functions [92]. In particular, it is required for the enzymatic reduction of oxidized glutathione, which is an essential part of the anti-oxidative systems, especially for the enzymatic activity of glutathione peroxidases [93]. NADPH is thought to be regenerated primarily via the oxidative pentose phosphate pathway [94]. Thus, down-regulation of the pentose phosphate pathway, which has been shown to occur in response to hypoxia [95], may lead to an increased susceptibility against oxidative stress in HO overexpressing cells.

An increased HO activity transiently augments the intracellular free iron pool [96] (or loosely bound or labile iron pool [97]) and leads to an increased ROS formation [74,98]. Although ROS stimulates ferritin, synthesis and sequestration of iron, the increased iron deposition may lead to iron overload, as has been shown following sustained HO overexpression [74,99]. Iron overload is generally associated with a higher risk of oxidative damage [100,101]. Under physiological conditions, iron is exported from cells of the reticuloendothelial system via ferroportin 1 (Fpn1) and redistributed in target tissues via transferrin. Particular signaling via toll like receptors (TLR), which results in degradation of the iron-exporter Fpn1 to withhold iron within the cells, may significantly contribute to critical iron overload (for review see: [102]) and increase the risk of iron-mediated oxidative damage [103,104].

A frequent complication in severe inflammatory conditions [20] and hypoxic episodes [105] is the displacement of iron from ferritin and other iron stores, by releasing chemically active ferrous molecules, which activate oxidative stress and cause tissue injury [106]. These iron species can be inactivated by applying iron chelators, such as desferrioxamine. The latter ameliorates post-ischemic reperfusion injury in the heart [107], kidney [108], intestine [109], and other tissues. Therefore, HO-mediated release of free iron from heme, as well as reductive mobilization of iron from ferritin (for review see: [110]) both contribute to an enlarged free iron pool facilitating iron-mediated ROS formation [111]. We and others have shown that HO could at least partially cause iron/ROS-mediated cellular dysfunction in conditions of severe inflammation [20]. In line with this observation, it has been shown that HO inhibition upon bile duct ligation attenuates rat liver fibrosis by reducing free iron levels [112].

Although mostly addressed, the HO-mediated effects are not solely explainable by the formation of the reaction products. HO also exerts its effects via the degradation of heme. It has been shown that an elevated HO activity leads to a measurable decrease of intracellular free heme levels [98]. Increased HO activity has been shown to diminish the levels of other heme proteins, such as cytochrome P450 enzymes, most likely via decreasing the intracellular heme pool [113]. Prolonged overexpression of HO-1 in cultured endothelial cells was accompanied by a time- and dose-dependent decrease in intracellular heme and an increase in the intracellular levels of cGMP [114]. In contrast, inhibition of HO, by applying the competitive HO-inhibitor tin-protoporphyrin, resulted in elevation of intracellular heme levels [115].

Generally, lowering heme levels is predominantly seen as an anti-oxidative defense strategy to limit heme-induced ROS formation, which is therefore considered as a beneficial action of HO. Heme, similarly to ferrous iron, represents a significant oxidative challenge and requires an efficient chaperoning/scavenging system for limiting formation of ROS. Of principal relevance, however, is the role of heme as a fundamental cell regulatory molecule. Besides controlling its own biosynthesis and export, it directly regulates gene expression, proliferation, cell function and differentiation, and is recognized as a master regulator of the cell fate (for review see: [79,116,117]). Therefore, by decreasing intracellular heme levels, HO is supposed to affect all those heme-controlled pathways as well. In the following sections, we will outline the roles of heme in the cell as a pro-oxidant and signaling molecule highlighting the pathways that are modulated by heme and consequently also by active HO.

## 3. Chemistry of Heme

In living organisms, iron-mediated catalytic processes are mainly exerted via heme, which contributes about 90% to all the iron present within the body. Heme is a chelating complex of iron with protoporphyrin IX, a ubiquitous compound among living organisms, and is essential for electron transfer reaction and reactions involving oxygen [79,118]. Heme exists in different modifications. The most common type of heme is heme b, which is bound non-covalently to proteins via coordination with the imidazole residue of histidine. In myoglobin and hemoglobin, heme b mediates oxygen storage and transport [119], while it plays a role in electron transfer reactions in the cytochrome P450 system, the xenobiotic detoxication enzymes of the endoplasmic reticulum. Apart from mixed function mono-oxygenases, peroxidases, catalases and synthases (e.g., nitric oxide synthase, NOS), cyclooxygenases, and several other mammalian proteins, including heme oxygenases itself, contain heme b. Heme b may dissociate from the holoprotein, which is facilitated upon oxidation of the central iron ion [120]. In contrast, heme c, the prosthetic group of cytochrome c, a part of the mitochondrial respiratory chain, is mainly prevented from dissociation by its covalent bond. HO cleaves both types of heme. However, the resulting biliverdin isoform cannot be converted into bilirubin [121].

Apart from hemoproteins, heme is present within the cell in the so-called labile heme pool, which primarily serves to maintain biosynthesis of hemoproteins. This heme is probably loosely bound to a set of proteins [118,122,123]. These proteins, similar to hemopexin and albumin within the intravascular space, scavenge heme and prevent its uncontrolled association with membranes and other lipophilic structures and subsequent induction of oxidative damage [124,125,126].

## 4. Pro-Oxidative Properties of Heme

Redox active heme iron can induce oxidative stress reactions, leading to the degradation of biomolecules, lipids, proteins and DNA [127]. These deleterious reactions can be prevented by the most prominent heme scavenger, hemopexin [128]. This suggests that the detrimental effects of heme are mainly associated with conditions of insufficient heme scavenging capacity, which can occur upon extensive hemolysis and the subsequent excessive release of heme into the blood. Un-scavenged heme in the circulation damages endothelial cells, oxidizes lipoproteins and activates systemic inflammatory responses [127]. Lipoproteins are predominant targets for heme-mediated oxidation in the blood. Free heme in plasma is capable of oxidizing low-density lipoprotein [129,130,131], damaging endothelial cells and leading to excessive immune stimulation and an elevated death rate of heme-handling macrophages [132,133,134,135]. These deleterious effects of heme can be diminished or even prevented by therapeutic administration of hemopexin [136].

The release of heme into the blood is considered as a driver of inflammation operating via activation of NF-κB or/and the nucleotide-binding domain and leucine rich repeat containing family, pyrin domain containing 3 (NLRP3) inflammasome [133,134]. Therefore, free heme appears to be an endogenous danger molecule [137]. However, it is still unclear whether effects of excessive heme administration described in a number of publications [132,133,134,135] are directly induced by heme or whether they are caused by secondary effects, such as release of DAMPs from cells damaged by heme-mediated oxidative stress reactions. The latter is supported by a recent observation, that heme pretreatment does not influence the inflammatory response induced by LPS in rats [138].

Intracellular accumulation of heme has also a pronounced deleterious effect. This occurs predominantly in erythropoietic precursor cells deficient in the heme exporter feline leukemia virus subgroup C receptor 1 (Flvcr1). Here accumulation of heme induces ROS-mediated cell death tentatively via apoptosis [139]. However, the impact of intracellular pro-oxidant reactions on cellular functions that are heme-catalysed is still poorly understood.

There are two mechanisms underlying pro-oxidant reactions of heme. The first mechanism, called the Fenton reaction [140], leads to the formation of highly toxic hydroxyl radicals (•OH) from the reaction of heme with hydrogen peroxide. The second mechanism is the formation of ferryl (Fe^4+^) and perferryl (Fe^5+^) species [140]. Both species have a very high oxidative potential [141] and react instantly with organic hydroperoxides [97].

**Figure 2:**
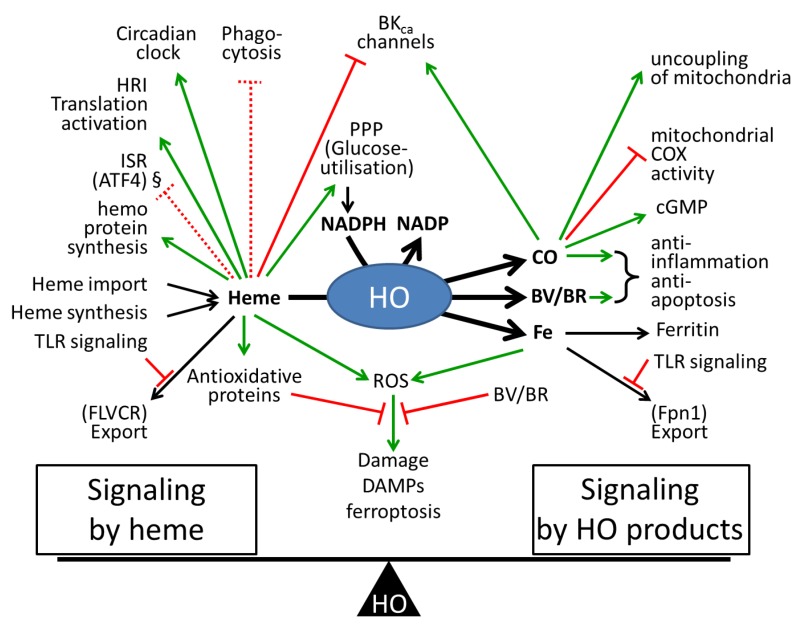
Role of HO as master regulator of cell physiology. Highly active HO shifts the balance from heme mediated signaling towards HO product mediated signaling. Low activity results in an increased heme mediated signaling. Black arrows indicate direct processes associated with HO activity. Green arrows indicate elicited effects; red blocks indicate inhibitory actions of the indicated compounds. Interactions occurring in specialized cells, such as erythroid cells, or macrophages, are indicated by dotted lines. §, integrated stress response (ISR) pathway is activated via ATF4 by heme deficiency, indicating that physiological heme levels repress ATF4 activation. Abbreviations: ATF4, Activating transcription factor 4; BK_Ca_, conductance Ca^2+^-activated K^+^ channels; BV/BR, biliverdin/bilirubin; cGMP, cyclic guanosine monophosphate, CO, carbon monoxide; COX, cytochrome c oxidase; DAMPs, danger associated molecular pattern; FLVCR, Feline leukemia virus subgroup C receptor; FPN1, ferroportin 1; PPP, pentose phosphates pathway; ROS, reactive oxygen species; TLR, Toll-like receptors.

Apart from the damage to biological structures, redox reactions of heme and non-heme free iron have recently been associated with ferroptosis, a newly discovered mechanism of programmed cell death [17,18,142]. The metabolic cornerstones of this death program include iron-dependent (lipoxygenase-mediated) generation of (phospho)-lipid hydro-peroxides and their reduction to alcohols by glutathione peroxidase 4 (GPX4) [143]. The latter prevents redox iron mediated decay of hydroperoxy-lipids to oxidatively truncated reactive electrophilic species [16] that attack critical protein targets and lead to cell death [144]. It is not clear yet whether intracellular heme or free ferrous iron pools predominantly contribute to ferroptosis, but since HO regulates the balance between heme and free ferrous ions, it may be an important trigger of cell death (see Figure 2).

Thus, the properties of heme allow interaction with high-molecular-weight biomolecules, resulting in oxidative damage and induction of inflammatory responses, or in execution of ferroptosis. As outlined above, chaperoning proteins, acting similarly to hemopexin, which operates as heme scavenger in the circulation, can largely prevent all these adverse effects of heme.

Apart from scavenging proteins, other endogenously produced molecules, the gaseous messengers nitric oxide (NO) and CO, can reduce the pro-oxidative capacity of heme. These two molecules have a high affinity for ferrous iron and they can diffuse through cell membranes. It has been shown that the formation of nitrosyl complexes upon reaction between NO with heme or free ferrous iron results in the loss of their catalytic activity [97]. Further, formation of nitrosyl iron complexes has been demonstrated to prevent iron-mediated lipid peroxidation, to preserve mitochondrial function [145], to induce cytoprotective effects in the skin [146], and in cancer cells [147]. More details about the action of NO and CO will be addressed in the following section.

## 5. Heme and NO/CO and the Resulting Reactions

The capability of NO to efficiently form nitrosyl-complexes has curiously been exploited from the tobacco industry, which used porcine blood-soaked filters to eliminate carcinogenic volatile nitroso-compounds from cigarette smoke. Impregnation with hemoglobin resulted in a 90% reduction of these compounds and simultaneously reduced the alveolar production of both NO and peroxynitrite (ONOO^−^) upon inhalation by 70% [148]. This property of heme is not limited to its localization in proteins. Also free heme acts as a scavenger or biological reservoir of NO within the body and is thereby able to modulate other signaling pathways via NO/CO messengers. NO donors reduce HO activity [149] and nitrosylated heme is not degradable by HO (own observation). Thus, enhanced NO generation may increase the cellular heme levels by inhibiting HO.

There is controversy in the literature regarding biological effects of NO derived from NOS; it has been shown that it may manifest both, beneficial and deleterious biological effects [150,151], which depend on the type of NOS (endothelial NOS, neuronal NOS, iNOS) and the type(s) of cells, which are exposed to NO [150]. Excessive release of NO may induce overwhelming vasodilation causing circulatory failure followed by multi-organ dysfunction and death. In contrast to NOS, nitrite, which acts as NO-donor upon reaction with deoxygenated hemoglobin, is mainly beneficial as summarized in several reviews [152,153].

## 6. Heme Modulating Cellular Pathways—From Transcription to Differentiation

### 6.1. Heme Regulating Its Degradation and the Anti-oxidative Defense 

In the redox reactions described in the previous sections, we considered direct interaction of heme with biomolecules yielding highly toxic reactive species. However, heme also contributes to the regulation of the redox homeostasis by affecting specific antioxidant systems indirectly. In nearly all mammalian cell types, heme induces its own degradation via the transcription factors Btb and Cnc Homology1 (Bach1). Activity of Bach1, a basic leucine zipper acting as a transcriptional repressor, is directly modulated by heme-binding [154,155]. Heterodimers of Bach1 with proteins of the Maf-related oncoprotein family bind to several target genes via the cis-acting sequence known as Maf Recognition Element (MARE)/Antioxidant Response Element (ARE) in their regulatory region [154,156,157]. Besides HO-1, ARE containing targets are several oxidative stress response genes, such as NAD(P)H quinone oxidoreductase 1, and glutathione S-transferases [158], as well as enzymes of the cellular glutathione biosynthetic pathway, such as glutamate-cysteine ligase catalytic subunit (GCLC), glutamate-cysteine ligase modifier (GCLM), and cystine/glutamate transporters (see Figure 2). Bach1 further regulates expression of globin genes, and key genes required for heme synthesis [154,157,159] (see Section 6.2), and finally genes related to the cell cycle and apoptosis [160].

Bach1 is a heme sensing protein and is activated by direct cooperative binding of heme at six heme binding motifs (HRM) [161,162]. HRM are short amino acid sequences that include a heme-coordination site. HRM are located on the surface of proteins which act as heme sensors [163]. In several proteins, such regulatory interaction with heme has been shown, amongst others the nuclear receptors Rev-erbα and Rev-erbβ, HO-2 (see also Section 6.3), and 5-aminolevulinate synthase. Recent evidence suggests the existence of these heme interaction sites in more proteins than so far known [164].

The binding of heme to the HRM of Bach1 results in its dissociation from AREs, its subsequent translocation into the cytoplasm, and further ubiquitination and degradation [165]. Except heme, other factors, such as cadmium, anchor Bach1 within the cytoplasm and thereby provoke the expression of ARE containing target genes [166]. Dislocation of Bach1 allows Maf to dimerize with another transcription factor, NF-E2-related factor 2 (Nrf2) [167]. Maf in conjunction with Nrf2 now acts as an activator of the transcription of ARE containing genes [155]. This shows that heme, by binding to Bach1, provokes a shift from gene repression to gene activation, realized by exchanging the Maf dimerization partner. Considering that Nrf2 is stabilized in the presence of electrophiles [168], which are generated under conditions of enhanced ROS formation, heme synergistically induces HO-1 together with several other proteins required for an enhanced ROS defense (see Figure 2). Heme has also been shown to regulate the transcription of the iron exporter Fpn1 through binding of Bach1 and Nrf2 at its ARE response element [169]. Fpn1 is required to expulse iron to avoid excessive iron accumulation under conditions of elevated heme supply (see Figure 2).

Thus, in addition to the direct pro-oxidant action of heme resulting in oxidative damage to biomolecules, it also enhances oxidant defense systems predominantly via upregulation of HO-1 and activation of the Nrf2 pathway. However, the activation of antioxidant systems takes time required for the gene expression. We assume that an acute increase in heme levels will have an immediate pro-oxidative effect and a delayed anti-oxidant effect, when respective pathways are activated and changes executed. However, active HO can slow down the activation of Nrf2, because it degrades heme. The latter can probably also account for the deleterious effects of chronic upregulation of HO mentioned above. In addition, HO shifts the iron balance from heme to ferrous iron, facilitates iron (re-)distribution via Fpn1 and transferrin to the body compartments with an increased demand for *de novo* hemoprotein synthesis. The highest amount of iron is used for heme biosynthesis in erythrocyte precursors, where heme plays a fundamental role supporting erythrocyte differentiation and maturation. This topic will be outlined in the following chapter.

### 6.2. Heme Regulating Erythrocyte Maturation and Differentiation

In healthy subjects, erythropoiesis continuously produces a sufficient amount of red blood cells to assure adequate tissue oxygenation (for recent reviews see: [170,171]. About 200 × 10^9^ erythrocytes are released each day from the bone marrow into the blood stream in healthy humans [172]. Erythropoiesis responds on the respective life conditions (i.e., pregnancy, altitude, physical exercise, infectious diseases, etc.) and can thus vary substantially. Red blood cell production critically depends on erythropoietin, which regulates survival, proliferation, and the rate of terminal maturation from erythroid progenitors [173]. A critical target of erythropoietin is GATA-1, a hematopoietic transcription factor, which orchestrates the expression of many target genes including those encoding erythrocyte constituents required for erythroid maturation [174]. The expression of hemoglobin subunits and heme biosynthetic enzymes, including the initial enzyme of the heme biosynthetic pathway, the erythroid-specific isoform 5-aminolevulinate synthase 2, occurs in a highly coordinated fashion. Both iron and heme are required in sufficient amounts to maintain erythroblast maturation. The intense demand of heme requires an accelerated iron import and heme biosynthesis, which therefore critically depends on iron availability. High levels of intracellular heme suppress the 5-aminolevulinate synthase 2, which is located in mitochondria [175]. Under heme deficiency, a part of GATA-1 target genes becomes GATA-1 insensitive. Among these genes are proteins that contribute to the building up of erythroid cell constituents [176]. The repressor Bach1 plays a particular role as a heme sensor for an important part of the erythroid precursor transcriptome. The coordination between heme synthesis, globin synthesis and other erythrocyte constituents is achieved via a complex interaction of GATA-1 and Bach1. GATA-1 induces expression of globin chains, 5-aminolevulinate synthase 2 and down-regulates Bach1 levels indirectly, via the increased heme synthesis. Heme, by binding to Bach1 amplifies GATA-1 driven globin gene expression, recruits Bach1 to the cytoplasm for degradation and diminishes Bach1-mediated repression. Bach1 is than replaced by the transcriptional activator p45 promoting enhanced gene transcription. High levels of heme repress 5-aminolevulinate synthase 2 at the level of mitochondrial import in erythroid progenitors. Hemoglobin assembly depends critically on the size of the cytosolic heme pool, which requires export of heme from mitochondria. Further, to protect erythroblasts from heme-mediated toxicity, excess heme must be expulsed from erythroid precursors [177,178]. Exportation of heme is achieved via the coordinated expression of Feline leukemia virus subgroup C receptor (FLVCR) 1, serving as a heme carrier [179]. FLVCR, which is encoded by the gene *SLC49A1*, contains binding sites for GATA1 [178] and a consensus binding motif for Bach1 [180]. Thus, via stimulating FLVCR expression, heme organizes its trafficking across membranes and expulsion. Heme deficiency results in a GATA-1 dependent accumulation of Bach1, repression of globin synthesis, and subsequently the activation of autophagy programs. Iron deficient mice develop anemia and show enhanced mitophagy in erythroblasts. Silencing Bach1 resulted in aggravation of anemia and de-repression of HO-1 [181], indicating that in erythroblasts HO, different to other cell types, is repressed under normal conditions to keep heme levels high.

These findings demonstrate that heme, by controlling GATA-1 function, rules an essential part of the erythroblast transcriptome, which regulates red blood cell differentiation and maturation. However, heme regulation occurs not only at transcriptional level, as outlined before, but additionally at post-transcriptional levels, which we describe in the following part.

It is well recognized that heme regulates the activity of eukaryote initiation factor 2alpha (eIF2α) kinase (HRI), which controls the translation of globin RNA and survival of erythroid precursors. Absence of heme leads to the activation of the kinase, which in turn phosphorylates eIF2α, and thereby leads to a general translation arrest [182]. Phosphorylation of eIF2α is also an integral part of the cellular stress response, which can be triggered by endoplasmic reticulum stress or enhanced oxidative stress [183]. Heme deficiency not only induces a general translation arrest, but simultaneously activates stress response pathways via ATF4 to prevent cytotoxicity elicited by accumulated globin chains [184]. Additionally, expression of ATF4 target genes is activated to maintain mitochondrial function in erythropoiesis in absence of heme [185]. Thus, heme at physiological levels prevents the activation of the integrated stress response (ISR) pathway via HRI and ATF4 (see Figure 2). All these findings demonstrate how regulative circuits involving heme ensure that this prosthetic group and the respective apo-proteins are available at the correct stoichiometric requirements. Further, heme prevents cytotoxic accumulation by inducing its own export, and prevents accumulation of excessive protein synthesis by activating the integrated stress response. The ubiquitous expression of Bach proteins suggests the existence of similar fine-tuned regulation circuits in other cell types manifesting high levels of hemoproteins.

### 6.3. Heme Regulating Energy Metabolism and Glucose Utilisation

Due to its important role as a prosthetic group, it is not surprising that heme regulates numerous cellular pathways at different levels. Heme is an essential component in the orchestration of oxygen-dependent energy metabolism, from glycolysis to mitochondrial respiration. Heme and glucose show an interesting interplay regulating each other’s metabolism in the liver. Expressions of gluconeogenic genes, such as phosphoenol pyruvate carboxykinase and the glucose-6-phosphatase are suppressed by heme, leading to decreased glucose excretion, when heme levels are high [186]. This suggests that heme deviates glucose to feed the pentose phosphate pathways needed to build up NADPH. Oxidative cleavage of heme and the subsequent reduction of BV to BR requires 4 mol NADPH per mol heme. Further, in liver cells, high levels of glucose maintain expression of the glycolytic key enzyme, 6-phosphofructo 2-kinase/fructose 2,6-bisphosphatase (PFKFB) 4 in a coordinated fashion with HO-2 [187]. PFKFBs are bifunctional enzymes with distinct kinase vs phosphatase activities that control the levels of fructose 2,6-bisphosphate by their predominantly executed reaction. Increased expression levels of PFKFB4 have been shown to accelerate production of fructose 2,6-bisphosphate from fructose 6-phosphate and to feed the glycolytic (anaerobic) ATP production [188]. This suggests that high HO activity favors anaerobic glycolysis and an inefficient energy provision. The tight association between heme metabolism and glucose utilization is also supported by the increased body weight, which HO-2 knockout mice display when compared to their wild type litter mates [187] (see Figure 2). Considering that the HO reaction consumes oxygen and NADPH, it can be expected that heme degradation compete with other oxygen consuming reactions, most of all the oxidative phosphorylation of mitochondria. HO further produces CO, which can interact with heme proteins, amongst others with mitochondrial hemoproteins. This strongly suggests that mitochondrial respiration and heme degradation are competing reactions. We will outline the findings underpinning this viewpoint in the following part.

The intracellular heme pool is central for mitochondrial function and oxygen metabolism [116,189]. For neuronal cells, which particularly depend on proper mitochondrial function, heme deficiency has been demonstrated as crucial factor leading to mitochondrial decay and degeneration, which is observed in aging [190] and Alzheimer’s disease [191]. Studies suggest that a decrease in heme levels prevents the assembly of complex IV, the terminal complex of the electron transport chain [192]. However, heme is not only required for supporting mitochondrial function by controlling the mitochondrial protein availability. It also regulates mitochondrial activity indirectly via the circadian clock [193].

The circadian clock is an oscillating molecular mechanism consisting of clock genes regulated as loops at the transcriptional and translational level, which regulates a number of cellular processes (for reviews see: [194,195]). Here, heme plays a central role. By binding to the nuclear receptors Rev-erbα and Rev-erbβ, heme represses target clock genes, including brain and muscle aryl hydrocarbon receptor nuclear translocator (ARNT)-like 1 (BMAL1), which together with Rev-erbα is responsible for the circadian oscillator mechanism [193,196]. Both, Rev-erbα expression and repression of its target gene BMAL1 are also required to control mitochondrial activity and enhance oxidative phosphorylation [197,198,199].

Thus, it appears that heme presence facilitates an efficient energy provision supporting mitochondrial function, while heme deficiency may shift the energy metabolism from oxidative phosphorylation towards anaerobic glycolysis. We have shown that acute inhibition of HO enhances mitochondrial respiration [200], suggesting that heme degradation is associated with a decrease in mitochondrial respiration. At least in yeast this role for heme has already been demonstrated: increased heme synthesis provoked a metabolic switch from fermentation to oxidative phosphorylation, while lowering heme levels favored anaerobic glycolysis, that is fermentation [201].

How could such a regulation be achieved in mammalian cells? First, HO, and particularly HO-1 protein turnover are regulated by the ubiquitin–proteasome system involving the really interesting new gene (RING)-type E3 ligase [202]. In susceptible cells, high glucose has been shown to modulate both, the ubiquitin/proteasome system, which is compromised [203], and the autophagic mechanism, especially mitophagy, which is enhanced [204]. Also, the HO reaction product CO may play a role. CO has been shown to inhibit mitochondrial respiration and thus oxygen consumption by different means [205]. Additionally, recent data suggest that CO also induces mitochondrial uncoupling [206,207], which is suggested to operate via the mitochondrial conductance Ca^2+^-activated K^+^ (BK_Ca_) channels [206]. CO mediated uncoupling has been shown to decrease ATP synthesis, which was associated with an accelerated glycolysis and lactate production in response to high glucose [207]. This effect may depend on the metabolic requirements of the cell type. In endothelial cells, despite inducing uncoupling, CO application resulted in inhibition of glycolysis [206], suggesting cell type specific regulation.

All these findings suggest that HO by two means, directly and acutely via enhanced production of CO, and indirectly and delayed by lowering intracellular heme levels and heme-mediated signaling, modulates mitochondrial oxygen consumption. Attenuation of mitochondrial respiration would also favor the heme breakdown, as the HO reaction requires 3 mol oxygen per mol heme [208]. In the view of all the findings discussed above it is tempting to speculate that heme degradation by active HO switches the cell metabolism from oxidative phosphorylation to anaerobic glycolysis. The heme degradation product CO, depending on cell type, could either support or counteract a heme induced metabolic switch. In metabolic syndrome we have previously shown that HO-1 modulates mitochondrial respiration by enhancing ROS production which inhibits adipocyte proliferation [209] and urge macrophages towards an inflammatory profile [21]. The capability to adapt the metabolism to the environment plays a particular important role for macrophages, enabling the execution of different functions. In the next chapter we will outline the role of heme regulating phenotypes and functions of macrophages.

### 6.4. Heme Regulating Macrophage Function

Macrophages are involved in recycling heme/heme iron from phagocytosed senescent erythrocytes [177] or the uptake of excess heme resulting from hemolysis. It has been shown that HO-1 deficiency in mammalians substantially reduce the activity of erythrophagocytosing macrophages and damage to spleen, liver and kidney [5]. Along with tissue-specific functions, macrophages are principal regulators of immune homeostasis. They can either promote inflammation, classically considered as M1-polarized macrophages, hallmarked by the production of pro-inflammatory cytokines and reactive oxygen species (ROS) or inhibit inflammation by producing anti-inflammatory cytokines, viewed as M2-polarized macrophages [210,211]. However, the rigid concept of M1/M2 polarization only insufficiently describes the mixed phenotypes, which macrophages express under different pathological conditions. This is explained by the high plasticity of macrophages as a result of the various factors, which fine-tune the macrophage’s phenotyp [212]. Heme and HO-1 appear to play an important role in modulating macrophage phenotype and function towards the anti-inflammatory M2-type [213,214]. The anti-inflammatory effects of IL-10 in macrophages were shown to be mediated by induction of HO-1 and CO generation [215,216]. Further, absence of HO-1 renders macrophages to become susceptible to heme mediated cell death, because HO-1 is required for cell protection from excess heme in erythrophagocytic macrophages and for attenuating the hepcidin mediated degradation of the iron exporter Fpn1 [217,218]. Heme regulates iron homeostasis in macrophages via the heme exporter Flvcr1 [219], and via Fpn1, required to export iron following HO-mediated heme degradation. In macrophages, Flvcr1 is considered as a kind of overflow valve that extrudes heme when the intracellular heme content reaches critical levels (for review see [220]). However, the inflammatory response to infection results in sequestration of heme and iron within cells. This mechanism has been shown to represent an efficient innate immune strategy to reduce the access of microbes to iron sources [221]. Inflammatory/infectious stimuli operating via stimulation of TLR polarize macrophages towards M1, which is associated with a decrease in Flvcr1 and Fpn1 expression [219]. Also pro-inflammatory cytokines, TNF-α or INF-γ or endogenous danger signals, released from damaged cells, have been shown lead to the differentiation into an M1 phenotype [222]. Thus, it can be assumed that under inflammatory conditions, heme levels rise within the macrophages, which contribute to the increased expression of HO-1.

Circulating free heme is considered as an endogenous stress or danger signal [137] termed ‘alarmin’, a compound that is able to recruit and activate innate immune cells, such as macrophages and dendritic cells and to promote adaptive immune response [223,224]. However, removal of pathogens is compromised when macrophages are subjected to excessive heme, or inhibition of HO. We have shown that heme decreases the phagocytic activity of macrophages challenged with bacteria [200]. Inhibition of phagocytosis is mediated by direct disruption of actin cytoskeletal dynamics [138], which are required to organize the engulfment of bacteria or host derived material [225]. These findings indicate that excessive release of heme as a consequence of hemolysis, or inhibition of HO, may compromise essential macrophage functions required for clearance of pathogenic material and thus resolution from diseases (see Figure 2).

## 7. Modulation of Heme-Mediated Signaling Pathways by HO Activity

Activity of HO is the result of the levels of functional protein from both isoforms, which are expressed at varying levels in most tissues. Besides the pure amount of protein, multiple regulatory mechanisms contribute to the overall activity. Changes in the catalytic activity can occur in both isoforms. We will outline in the following part what is known for either HO isoform. Regulation of HO activity at the protein level has mostly been described for HO-2. However, loss of function can also occur by cleavage and translocation into other compartments. Proteolytic cleavage of the ER-membrane anchor of HO-1 has been shown to mediate HO-1 translocation into the nucleus [226]. Such translocation has been suggested to go along with a changed function of HO-1, since heme degradation may not occur in the nucleus. It is instead assumed that truncated HO-1 may control gene expression. It has been shown that a catalytically inactive mutant HO-1 protein down-regulated the transcription factor NF-κB [226].

Activity of HO-2, in contrast to that of HO-1, appears to be regulated by multiple modifications. In most species, multiple transcripts encoding HO-2 have been reported, which may undertake cell-specific roles in development [227]. Similarly to Bach1, HO-2 contains HRMs in the C-terminus, which regulate activity and stability of the respective protein [228]. Additionally, the cysteine residues of these HRMs form a redox sensitive thiol/disulfide switching mechanism, suggested to act as cellular redox sensor [229]. This thiol/disulfide redox switch in HO-2 serves to modulate the affinity to its substrate heme [230]. The oxidative formation of disulfide bonds in HO-2 is associated with an increased heme affinity and CO generation. Thus heme is able to directly regulate the activity of HO-2. Both increased CO generation or decreased heme availability operate as signaling mechanism for activating BK_Ca_ channels involved in neural firing, muscle contraction, hearing, and vascular tone modulation [44]. These channels are located in the plasma membrane and activated by depolarization and the increase in intracellular Ca^2+^. Heme inhibits, while CO activates opening of the BK_Ca_ channels (see Figure 1 and Figure 2).

Apart from the exiting regulation by heme, activity of HO-2 can be influenced by several chemical modifications. Phosphorylation of neuronal HO-2 has been described to enhance activity of the enzyme and is considered an important mechanism for quickly increasing CO generation for neuronal signaling [231]. Further, Ca^2+^ mobilizing agents, such as ionomycin and glutamate, stimulate endogenous HO-2 activity, which is induced via a calmodulin binding site [232]. Modifications inhibiting HO-enzyme activity have also been described. It has been suggested that both isoforms, HO-1 together with HO-2, may form a complex, which displays a decreased HO activity [233]. Also, oxidation or nitrosylation of HO protein is associated with a decrease or a loss of function [234,235], possibly via interaction with the heme moiety.

Obviously, each modulation of the overall activity of HO enzymes will result in a transient imbalance of the ratio between heme and the HO products (see Figure 2). There is a certain capability to substitute the loss of one isoform by the other. Inhibition of HO frequently results in a subsequent super-induction of HO-1 at the transcriptional level, as has been frequently reported [236]. It has further been shown that HO-1 expression is induced when HO-2 is lost [115]. Genetic deletion of HO-2 lead to a compensatory upregulation of HO-1 and an enhanced HO activity. Also, (ii) knockout of HO (mostly done for HO-1) is likely to result in a compensatory adaptation of metabolically relevant enzymes to keep homeostasis, as was already shown in several cases [237]. However, it appears that HO-1, although it is exquisitely inducible, cannot fully overtake the role of HO-2. It has been shown that HO-1 derived activity does not protect neurons with a deleted HO-2 isoform [238].

These data demonstrate that the cellular HO activity may be much more dynamic than can be inferred from the expression levels of both enzymes. It can thus be assumed that the ratio between heme and HO products is subjected to the same dynamic variations and that consequently an inhibited HO activity enhances heme-controlled pathways, while an over-expressed HO activity enhances those controlled by the HO products (see Figure 2).

## 8. Conclusions

HO is a unique enzyme, which apart from its primary function, recovery of iron from heme for synthetic purposes, also regulates complex cascades of intracellular physiological signaling pathways. HO regulates signal transduction by the release of BV, iron, or the short living product, CO, but also by its substrate, heme. Intracellular heme amount results from fluxes across membranes, synthesis and consumption for assembly of hemoproteins, and its degradation by HO. Active HO leads to the activation of signaling events governed by its products, which occur on the account of pathways activated by the HO substrate, heme. Vice versa, a deceleration of the HO reaction will enhance heme mediated signaling, while lowering the effects elicited by heme degradation products. Due to only limited redundancy or cooperation between both sets of signaling events, modulation of HO activity has the potential to switch between different metabolic programs. However, dysregulation of HO, such over-activation or prolonged inhibition, will result in loss of balance of these two metabolic master programs. One example is the compromised heme/iron distribution, associated with chronic inflammation, which by itself is a potent inducer of HO-1. Thus, therapeutic targeting of HO should take into account the complex regulatory role of HO, which not only influences the network regulated by its products, but also that modulated by heme.

## References

[1] Munoz-Sanchez J., Chanez-Cardenas M.E. (2014). A review on hemeoxygenase-2: Focus on cellular protection and oxygen response. Oxid. Med. Cell Longev..

[2] McCoubrey W.K., Huang T.J., Maines M.D. (1997). Isolation and characterization of a cDNA from the rat brain that encodes hemoprotein heme oxygenase-3. Eur. J. Biochem..

[3] Poss K.D., Thomas M.J., Ebralidze A.K., O’Dell T.J., Tonegawa S. (1995). Hippocampal long-term potentiation is normal in heme oxygenase-2 mutant mice. Neuron.

[4] Poss K.D., Tonegawa S. (1997). Heme oxygenase 1 is required for mammalian iron reutilization. Proc. Natl. Acad. Sci. USA.

[5] Kovtunovych G., Eckhaus M.A., Ghosh M.C., Ollivierre-Wilson H., Rouault T.A. (2010). Dysfunction of the heme recycling system in heme oxygenase 1-deficient mice: Effects on macrophage viability and tissue iron distribution. Blood.

[6] Radhakrishnan N., Yadav S.P., Sachdeva A., Wada T., Yachie A. (2011). An interesting tetrad of asplenia, inflammation, hemolysis, and nephritis. Pediatr. Hematol. Oncol..

[7] Poss K.D., Tonegawa S. (1997). Reduced stress defense in heme oxygenase 1-deficient cells. Proc. Natl. Acad. Sci. USA.

[8] Ewing J.F., Maines M.D. (1997). Histochemical localization of heme oxygenase-2 protein and mRNA expression in rat brain. Brain Res. Brain Res. Protoc..

[9] Boehning D., Snyder S.H. (2003). Novel neural modulators. Annu. Rev. Neurosci..

[10] Mancuso C. (2004). Heme oxygenase and its products in the nervous system. Antioxid. Redox. Signal..

[11] Aztatzi-Santillan E., Nares-Lopez F.E., Marquez-Valadez B., Aguilera P., Chanez-Cardenas M.E. (2010). The protective role of heme oxygenase-1 in cerebral ischemia. Cent. Nerv. Syst. Agents Med. Chem..

[12] Camara N.O., Soares M.P. (2005). Heme oxygenase-1 (HO-1), a protective gene that prevents chronic graft dysfunction. Free Radic. Biol. Med..

[13] Li S., Fujino M., Takahara T., Li X.K. (2019). Protective role of heme oxygenase-1 in fatty liver ischemia-reperfusion injury. Med Mol. Morphol..

[14] Otterbein L.E., Soares M.P., Yamashita K., Bach F.H. (2003). Heme oxygenase-1: Unleashing the protective properties of heme. Trends Immunol..

[15] Soares M.P., Brouard S., Smith R.N., Bach F.H. (2001). Heme oxygenase-1, a protective gene that prevents the rejection of transplanted organs. Immunol. Rev..

[16] Stoyanovsky D.A., Tyurina Y.Y., Shrivastava I., Bahar I., Tyurin V.A., Protchenko O., Jadhav S., Bolevich S.B., Kozlov A.V., Vladimirov Y.A. (2019). Iron catalysis of lipid peroxidation in ferroptosis: Regulated enzymatic or random free radical reaction?. Free Radic. Biol. Med..

[17] Conrad M., Kagan V.E., Bayir H., Pagnussat G.C., Head B., Traber M.G., Stockwell B.R. (2018). Regulation of lipid peroxidation and ferroptosis in diverse species. Genes Dev..

[18] Dixon S.J., Lemberg K.M., Lamprecht M.R., Skouta R., Zaitsev E.M., Gleason C.E., Patel D.N., Bauer A.J., Cantley A.M., Yang W.S. (2012). Ferroptosis: An iron-dependent form of nonapoptotic cell death. Cell.

[19] Bernardini C., Grilli E., Duvigneau J.C., Zannoni A., Tugnoli B., Gentilini F., Bertuzzi T., Spinozzi S., Camborata C., Bacci M.L. (2014). Cellular stress marker alteration and inflammatory response in pigs fed with an ochratoxin contaminated diet. Res. Vet. Sci..

[20] Duvigneau J.C., Piskernik C., Haindl S., Kloesch B., Hartl R.T., Huttemann M., Lee I., Ebel T., Moldzio R., Gemeiner M. (2008). A novel endotoxin-induced pathway: Upregulation of heme oxygenase 1, accumulation of free iron, and free iron-mediated mitochondrial dysfunction. Lab. Invest..

[21] Jais A., Einwallner E., Sharif O., Gossens K., Lu T.T., Soyal S.M., Medgyesi D., Neureiter D., Paier-Pourani J., Dalgaard K. (2014). Heme oxygenase-1 drives metaflammation and insulin resistance in mouse and man. Cell.

[22] Postl A., Zifko C., Hartl R.T., Ebel T., Miller I., Moldzio R., Redl H., Kozlov A.V., Bahrami S., Duvigneau J.C. (2011). Transient increase of free iron in rat livers following hemorrhagic-traumatic shock and reperfusion is independent of heme oxygenase 1 upregulation. Shock.

[23] Ryter S.W., Alam J., Choi A.M. (2006). Heme oxygenase-1/carbon monoxide: From basic science to therapeutic applications. Physiol Rev..

[24] Belcher J.D., Chen C., Nguyen J., Abdulla F., Zhang P., Nguyen H., Nguyen P., Killeen T., Miescher S.M., Brinkman N. (2018). Haptoglobin and hemopexin inhibit vaso-occlusion and inflammation in murine sickle cell disease: Role of heme oxygenase-1 induction. PLoS ONE.

[25] Sassa S. (2004). Why heme needs to be degraded to iron, biliverdin IXalpha, and carbon monoxide?. Antioxid. Redox. Signal..

[26] Stocker R. (2004). Antioxidant activities of bile pigments. Antioxid. Redox. Signal..

[27] Maghzal G.J., Leck M.C., Collinson E., Li C., Stocker R. (2009). Limited role for the bilirubin-biliverdin redox amplification cycle in the cellular antioxidant protection by biliverdin reductase. J. Biol. Chem..

[28] Basuroy S., Bhattacharya S., Leffler C.W., Parfenova H. (2009). Nox4 NADPH oxidase mediates oxidative stress and apoptosis caused by TNF-alpha in cerebral vascular endothelial cells. Am. J. Physiol. Cell Physiol..

[29] Chen J., Tu Y., Connolly E.C., Ronnett G.V. (2005). Heme oxygenase-2 protects against glutathione depletion-induced neuronal apoptosis mediated by bilirubin and cyclic GMP. Curr. Neurovasc. Res..

[30] Jangi S., Otterbein L., Robson S. (2013). The molecular basis for the immunomodulatory activities of unconjugated bilirubin. Int. J Biochem. Cell Biol..

[31] Longhi M.S., Vuerich M., Kalbasi A., Kenison J.E., Yeste A., Csizmadia E., Vaughn B., Feldbrugge L., Mitsuhashi S., Wegiel B. (2017). Bilirubin suppresses Th17 immunity in colitis by upregulating CD39. JCI Insight.

[32] Valaskova P., Dvorak A., Lenicek M., Zizalova K., Kutinova-Canova N., Zelenka J., Cahova M., Vitek L., Muchova L. (2019). Hyperbilirubinemia in Gunn Rats is Associated with Decreased Inflammatory Response in LPS-Mediated Systemic Inflammation. Int. J. Mol. Sci..

[33] Wang W.W., Smith D.L., Zucker S.D. (2004). Bilirubin inhibits iNOS expression and NO production in response to endotoxin in rats. Hepatology.

[34] Gordon D.M., Blomquist T.M., Miruzzi S.A., McCullumsmith R., Stec D.E., Hinds T.D. (2019). RNA sequencing in human HepG2 hepatocytes reveals PPAR-alpha mediates transcriptome responsiveness of bilirubin. Physiol. Genom..

[35] Hinds T.D., Adeosun S.O., Alamodi A.A., Stec D.E. (2016). Does bilirubin prevent hepatic steatosis through activation of the PPARalpha nuclear receptor?. Med. Hypotheses.

[36] Stec D.E., John K., Trabbic C.J., Luniwal A., Hankins M.W., Baum J., Hinds T.D. (2016). Bilirubin Binding to PPARalpha Inhibits Lipid Accumulation. PLoS ONE.

[37] Chinetti G., Fruchart J.C., Staels B. (2000). Peroxisome proliferator-activated receptors (PPARs): Nuclear receptors at the crossroads between lipid metabolism and inflammation. Inflamm. Res..

[38] Wahli W. (2012). and Michalik, L. PPARs at the crossroads of lipid signaling and inflammation. Trends Endocrinol. Metab..

[39] Adeosun S.O., Gordon D.M., Weeks M.F., Moore K.H., Hall J.E., Hinds T.D., Stec D.E. (2018). Loss of biliverdin reductase-A promotes lipid accumulation and lipotoxicity in mouse proximal tubule cells. Am. J. Physiol. Ren. Physiol..

[40] Hinds T.D., Burns K.A., Hosick P.A., McBeth L., Nestor-Kalinoski A., Drummond H.A., Alamodi A.A., Hankins M.W., Vanden Heuvel J.P., Stec D.E. (2016). Biliverdin Reductase A Attenuates Hepatic Steatosis by Inhibition of Glycogen Synthase Kinase (GSK) 3beta Phosphorylation of Serine 73 of Peroxisome Proliferator-activated Receptor (PPAR) alpha. J. Biol. Chem..

[41] Cimini F.A., Arena A., Barchetta I., Tramutola A., Ceccarelli V., Lanzillotta C., Fontana M., Bertoccini L., Leonetti F., Capoccia D. (2019). Reduced biliverdin reductase-A levels are associated with early alterations of insulin signaling in obesity. Biochim. Biophys. Acta Mol. Basis. Dis..

[42] O’Brien L., Hosick P.A., John K., Stec D.E., Hinds T.D. (2015). Biliverdin reductase isozymes in metabolism. Trends Endocrinol. Metab..

[43] Brann D.W., Bhat G.K., Lamar C.A., Mahesh V.B. (1997). Gaseous transmitters and neuroendocrine regulation. Neuroendocrinology.

[44] Telezhkin V., Brazier S.P., Mears R., Muller C.T., Riccardi D., Kemp P.J. (2011). Cysteine residue 911 in C-terminal tail of human BK(Ca)alpha channel subunit is crucial for its activation by carbon monoxide. Pflug. Arch..

[45] Bernabeu R., Princ F., de Stein M.L., Fin C., Juknat A.A., Batile A., Izquierdo I., Medina J.H. (1995). Evidence for the involvement of hippocampal CO production in the acquisition and consolidation of inhibitory avoidance learning. Neuroreport.

[46] Motterlini R., Foresti R. (2017). Biological signaling by carbon monoxide and carbon monoxide-releasing molecules. Am. J. Physiol. Cell Physiol..

[47] Buechler C., Pohl R., Aslanidis C. (2017). Pro-Resolving Molecules-New Approaches to Treat Sepsis?. Int. J. Mol. Sci..

[48] Chen J. (2014). Heme oxygenase in neuroprotection: From mechanisms to therapeutic implications. Rev. Neurosci..

[49] Dennery P.A. (2014). Signaling function of heme oxygenase proteins. Antioxid. Redox. Signal..

[50] Lee H., Choi Y.K. (2018). Regenerative Effects of Heme Oxygenase Metabolites on Neuroinflammatory Diseases. Int. J. Mol. Sci..

[51] Otterbein L.E., Choi A.M. (2000). Heme oxygenase: Colors of defense against cellular stress. Am. J. Physiol. Lung Cell. Mol. Physiol..

[52] Li V.G., Tibullo D., Vanella L., Giallongo C., Di R.F., Forte S., Di R.M., Signorelli S.S., Barbagallo I. (2017). The Heme Oxygenase System in Hematological Malignancies. Antioxid. Redox. Signal..

[53] Podkalicka P., Mucha O., Jozkowicz A., Dulak J., Loboda A. (2018). Heme oxygenase inhibition in cancers: Possible tools and targets. Contemp. Oncol. (Pozn. ).

[54] Chau L.Y. (2015). Heme oxygenase-1: Emerging target of cancer therapy. J. Biomed. Sci..

[55] Loboda A., Jozkowicz A., Dulak J. (2015). HO-1/CO system in tumor growth, angiogenesis and metabolism —Targeting HO-1 as an anti-tumor therapy. Vascul. Pharmacol..

[56] Nitti M., Piras S., Marinari U.M., Moretta L., Pronzato M.A., Furfaro A.L. (2017). HO-1 Induction in Cancer Progression: A Matter of Cell Adaptation. Antioxidants.

[57] Lin Q.S., Weis S., Yang G., Zhuang T., Abate A., Dennery P.A. (2008). Catalytic inactive heme oxygenase-1 protein regulates its own expression in oxidative stress. Free Radic. Biol. Med..

[58] Frank J., Lornejad-Schafer M.R., Schoffl H., Flaccus A., Lambert C., Biesalski H.K. (2007). Inhibition of heme oxygenase-1 increases responsiveness of melanoma cells to ALA-based photodynamic therapy. Int. J. Oncol..

[59] Raffaele M., Pittala V., Zingales V., Barbagallo I., Salerno L., Li V.G., Romeo G., Carota G., Sorrenti V., Vanella L. (2019). Heme Oxygenase-1 Inhibition Sensitizes Human Prostate Cancer Cells towards Glucose Deprivation and Metformin-Mediated Cell Death. Int. J. Mol. Sci..

[60] Salerno L., Romeo G., Modica M.N., Amata E., Sorrenti V., Barbagallo I., Pittala V. (2017). Heme oxygenase-1: A new druggable target in the management of chronic and acute myeloid leukemia. Eur. J. Med. Chem..

[61] Mucha O., Podkalicka P., Czarnek M., Biela A., Mieczkowski M., Kachamakova-Trojanowska N., Stepniewski J., Jozkowicz A., Dulak J., Loboda A. (2018). Pharmacological versus genetic inhibition of heme oxygenase-1—the comparison of metalloporphyrins, shRNA and CRISPR/Cas9 system. Acta Biochim. Pol..

[62] Hasegawa Y., Takano T., Miyauchi A., Matsuzuka F., Yoshida H., Kuma K., Amino N. (2002). Decreased expression of glutathione peroxidase mRNA in thyroid anaplastic carcinoma. Cancer Lett..

[63] Hasegawa Y., Takano T., Miyauchi A., Matsuzuka F., Yoshida H., Kuma K., Amino N. (2003). Decreased expression of catalase mRNA in thyroid anaplastic carcinoma. Jpn. J. Clin. Oncol..

[64] Sato K., Ito K., Kohara H., Yamaguchi Y., Adachi K., Endo H. (1992). Negative regulation of catalase gene expression in hepatoma cells. Mol. Cell Biol..

[65] Takeuchi T., Nakamura S., Kayasuga A., Isa S., Sato K. (2000). Multiple elements for negative regulation of the rat catalase gene expression in dedifferentiated hepatoma cells. J. Biochem..

[66] Sahoo S.K., Sawa T., Fang J., Tanaka S., Miyamoto Y., Akaike T., Maeda H. (2002). Pegylated zinc protoporphyrin: A water-soluble heme oxygenase inhibitor with tumor-targeting capacity. Bioconjug. Chem..

[67] Lv X., Song D.M., Niu Y.H., Wang B.S. (2016). Inhibition of heme oxygenase-1 enhances the chemosensitivity of laryngeal squamous cell cancer Hep-2 cells to cisplatin. Apoptosis.

[68] Liu Y.S., Li H.S., Qi D.F., Zhang J., Jiang X.C., Shi K., Zhang X.J., Zhang X.H. (2014). Zinc protoporphyrin IX enhances chemotherapeutic response of hepatoma cells to cisplatin. World, J. Gastroenterol..

[69] Fang J., Sawa T., Akaike T., Akuta T., Sahoo S.K., Khaled G., Hamada A., Maeda H. (2003). In vivo antitumor activity of pegylated zinc protoporphyrin: Targeted inhibition of heme oxygenase in solid tumor. Cancer Res..

[70] Fang J., Tsukigawa K., Liao L., Yin H., Eguchi K., Maeda H. (2016). Styrene-maleic acid-copolymer conjugated zinc protoporphyrin as a candidate drug for tumor-targeted therapy and imaging. J. Drug Target.

[71] Salerno L., Pittala V., Romeo G., Modica M.N., Marrazzo A., Siracusa M.A., Sorrenti V., Di G.C., Vanella L., Parayath N.N. (2015). Novel imidazole derivatives as heme oxygenase-1 (HO-1) and heme oxygenase-2 (HO-2) inhibitors and their cytotoxic activity in human-derived cancer cell lines. Eur. J. Med. Chem..

[72] Greish K.F., Salerno L., Al Z.R., Amata E., Modica M.N., Romeo G., Marrazzo A., Prezzavento O., Sorrenti V., Rescifina A. (2018). Novel Structural Insight into Inhibitors of Heme Oxygenase-1 (HO-1) by New Imidazole-Based Compounds: Biochemical and In Vitro Anticancer Activity Evaluation. Molecules.

[73] Appleton S.D., Chretien M.L., McLaughlin B.E., Vreman H.J., Stevenson D.K., Brien J.F., Nakatsu K., Maurice D.H., Marks G.S. (1999). Selective inhibition of heme oxygenase, without inhibition of nitric oxide synthase or soluble guanylyl cyclase, by metalloporphyrins at low concentrations. Drug Metab. Dispos..

[74] Suttner D.M., Dennery P.A. (1999). Reversal of HO-1 related cytoprotection with increased expression is due to reactive iron. FASEB J..

[75] Chang L.C., Chiang S.K., Chen S.E., Yu Y.L., Chou R.H., Chang W.C. (2018). Heme oxygenase-1 mediates BAY 11-7085 induced ferroptosis. Cancer Lett..

[76] Kwon M.Y., Park E., Lee S.J., Chung S.W. (2015). Heme oxygenase-1 accelerates erastin-induced ferroptotic cell death. Oncotarget.

[77] Tronel C., Rochefort G.Y., Arlicot N., Bodard S., Chalon S., Antier D. (2013). Oxidative stress is related to the deleterious effects of heme oxygenase-1 in an in vivo neuroinflammatory rat model. Oxid. Med. Cell Longev..

[78] Hopper C.P., Meinel L., Steiger C., Otterbein L.E. (2018). Where is the Clinical Breakthrough of Heme Oxygenase-1 / Carbon Monoxide Therapeutics?. Curr. Pharm. Des..

[79] Ryter S.W., Tyrrell R.M. (2000). The heme synthesis and degradation pathways: Role in oxidant sensitivity. Heme oxygenase has both pro- and antioxidant properties. Free Radic. Biol. Med..

[80] Tsuchihashi S., Livhits M., Zhai Y., Busuttil R.W., Araujo J.A., Kupiec-Weglinski J.W. (2006). Basal rather than induced heme oxygenase-1 levels are crucial in the antioxidant cytoprotection. J. Immunol..

[81] Ohnishi M., Katsuki H., Unemura K., Izumi Y., Kume T., Takada-Takatori Y., Akaike A. (2010). Heme oxygenase-1 contributes to pathology associated with thrombin-induced striatal and cortical injury in organotypic slice culture. Brain Res..

[82] Song L., Song W., Schipper H.M. (2007). Astroglia overexpressing heme oxygenase-1 predispose co-cultured PC12 cells to oxidative injury. J. Neurosci. Res..

[83] Andrews M., Leiva E., Arredondo-Olguin M. (2016). Short repeats in the heme oxygenase 1 gene promoter is associated with increased levels of inflammation, ferritin and higher risk of type-2 diabetes mellitus. J. Trace Elem. Med. Biol..

[84] Mishra M., Ndisang J.F. (2014). A critical and comprehensive insight on heme oxygenase and related products including carbon monoxide, bilirubin, biliverdin and ferritin in type-1 and type-2 diabetes. Curr. Pharm. Des..

[85] Chen Q.Y., Wang G.G., Li W., Jiang Y.X., Lu X.H., Zhou P.P. (2016). Heme Oxygenase-1 Promotes Delayed Wound Healing in Diabetic Rats. J. Diabetes Res..

[86] Pagnin E., Maiolino G., Calo L.A. (2016). Heme oxygenase-1 in type 2 diabetes: From cell first-line defense to early marker of diabetic nephropathy. Minerva Med.

[87] Tsuji M.H., Yanagawa T., Iwasa S., Tabuchi K., Onizawa K., Bannai S., Toyooka H., Yoshida H. (1999). Heme oxygenase-1 expression in oral squamous cell carcinoma as involved in lymph node metastasis. Cancer Lett..

[88] Tsai J.R., Wang H.M., Liu P.L., Chen Y.H., Yang M.C., Chou S.H., Cheng Y.J., Yin W.H., Hwang J.J., Chong I.W. (2012). High expression of heme oxygenase-1 is associated with tumor invasiveness and poor clinical outcome in non-small cell lung cancer patients. Cell Oncol..

[89] Noh S.J., Bae J.S., Jamiyandorj U., Park H.S., Kwon K.S., Jung S.H., Youn H.J., Lee H., Park B.H., Chung M.J. (2013). Expression of nerve growth factor and heme oxygenase-1 predict poor survival of breast carcinoma patients. BMC Cancer.

[90] Wang T.Y., Liu C.L., Chen M.J., Lee J.J., Pun P.C., Cheng S.P. (2015). Expression of haem oxygenase-1 correlates with tumour aggressiveness and BRAF V600E expression in thyroid cancer. Histopathology.

[91] Zhao Z., Xu Y., Lu J., Xue J., Liu P. (2018). High expression of HO-1 predicts poor prognosis of ovarian cancer patients and promotes proliferation and aggressiveness of ovarian cancer cells. Clin. Transl. Oncol..

[92] Pollak N., Dolle C., Ziegler M. (2007). The power to reduce: Pyridine nucleotides--small molecules with a multitude of functions. Biochem. J..

[93] Flohe L. (1978). Glutathione peroxidase: Fact and fiction. Ciba Found. Symp..

[94] Lunt S.Y., Vander Heiden M.G. (2011). Aerobic glycolysis: Meeting the metabolic requirements of cell proliferation. Annu. Rev. Cell Dev. Biol..

[95] Kathagen-Buhmann A., Schulte A., Weller J., Holz M., Herold-Mende C., Glass R., Lamszus K. (2016). Glycolysis and the pentose phosphate pathway are differentially associated with the dichotomous regulation of glioblastoma cell migration versus proliferation. Neuro-Oncology.

[96] Sergent O., Tomasi A., Ceccarelli D., Masini A., Nohl H., Cillard P., Cillard J., Vladimirov Y.A., Kozlov A.V. (2005). Combination of iron overload plus ethanol and ischemia alone give rise to the same endogenous free iron pool. Biometals.

[97] Kagan V.E., Kozlov A.V., Tyurina Y.Y., Shvedova A.A., Yalowich J.C. (2001). Antioxidant mechanisms of nitric oxide against iron-catalyzed oxidative stress in cells. Antioxid. Redox. Signal..

[98] Li C., Lonn M.E., Xu X., Maghzal G.J., Frazer D.M., Thomas S.R., Halliwell B., Richardson D.R., Anderson G.J., Stocker R. (2012). Sustained expression of heme oxygenase-1 alters iron homeostasis in nonerythroid cells. Free Radic. Biol. Med.

[99] Lamb N.J., Quinlan G.J., Mumby S., Evans T.W., Gutteridge J.M. (1999). Haem oxygenase shows pro-oxidant activity in microsomal and cellular systems: Implications for the release of low-molecular-mass iron. Biochem. J..

[100] Fibach E., Rachmilewitz E.A. (2017). Iron overload in hematological disorders. Presse Med..

[101] Puntarulo S. (2005). Iron, oxidative stress and human health. Mol. Asp. Med..

[102] Ginzburg Y.Z. (2019). Hepcidin-ferroportin axis in health and disease. Vitam. Horm..

[103] Hofer T., Perry G. (2016). Nucleic acid oxidative damage in Alzheimer’s disease-explained by the hepcidin-ferroportin neuronal iron overload hypothesis?. J. Trace Elem. Med. Biol..

[104] Kew M.C. (2009). Hepatic iron overload and hepatocellular carcinoma. Cancer Lett..

[105] Weidinger A., Dungel P., Perlinger M., Singer K., Ghebes C., Duvigneau J.C., Mullebner A., Schafer U., Redl H., Kozlov A.V. (2013). Experimental data suggesting that inflammation mediated rat liver mitochondrial dysfunction results from secondary hypoxia rather than from direct effects of inflammatory mediators. Front. Physiol..

[106] White B.C., Krause G.S., Aust S.D., Eyster G.E. (1985). Postischemic tissue injury by iron-mediated free radical lipid peroxidation. Ann. Emerg. Med..

[107] Badylak S.F., Simmons A., Turek J., Babbs C.F. (1987). Protection from reperfusion injury in the isolated rat heart by postischaemic deferoxamine and oxypurinol administration. Cardiovasc. Res..

[108] Paller M.S., Hedlund B.E. (1988). Role of iron in postischemic renal injury in the rat. Kidney Int..

[109] Hernandez L.A., Grisham M.B., Granger D.N. (1987). A role for iron in oxidant-mediated ischemic injury to intestinal microvasculature. Am. J. Physiol..

[110] Bou-Abdallah F., Paliakkara J.J., Melman G., Melman A. (2018). Reductive Mobilization of Iron from Intact Ferritin: Mechanisms and Physiological Implication. Pharmaceuticals.

[111] Jomova K., Valko M. (2011). Importance of iron chelation in free radical-induced oxidative stress and human disease. Curr. Pharm. Des..

[112] Wang Q.M., Du J.L., Duan Z.J., Guo S.B., Sun X.Y., Liu Z. (2013). Inhibiting heme oxygenase-1 attenuates rat liver fibrosis by removing iron accumulation. World J. Gastroenterol..

[113] Reichen J., Hoilien C., Sheldon G.F., Kirshenbaum G. (1983). A novel method for continuous monitoring of bilirubin production in unstressed rats. Am. J. Physiol..

[114] Abraham N.G., Quan S., Mieyal P.A., Yang L., Burke-Wolin T., Mingone C.J., Goodman A.I., Nasjletti A., Wolin M.S. (2002). Modulation of cGMP by human HO-1 retrovirus gene transfer in pulmonary microvessel endothelial cells. Am. J. Physiol. Lung Cell. Mol. Physiol..

[115] Ding Y., Zhang Y.Z., Furuyama K., Ogawa K., Igarashi K., Shibahara S. (2006). Down-regulation of heme oxygenase-2 is associated with the increased expression of heme oxygenase-1 in human cell lines. FEBS J..

[116] Mense S.M., Zhang L. (2006). Heme: A versatile signaling molecule controlling the activities of diverse regulators ranging from transcription factors to MAP kinases. Cell Res..

[117] Furuyama K., Kaneko K., Vargas P.D. (2007). Heme as a magnificent molecule with multiple missions: Heme determines its own fate and governs cellular homeostasis. Tohoku J. Exp. Med..

[118] Ponka P. (1997). Tissue-specific regulation of iron metabolism and heme synthesis: Distinct control mechanisms in erythroid cells. Blood.

[119] Reeder B.J. (2010). The redox activity of hemoglobins: From physiologic functions to pathologic mechanisms. Antioxid. Redox. Signal..

[120] Kassa T., Jana S., Meng F., Alayash A.I. (2016). Differential heme release from various hemoglobin redox states and the upregulation of cellular heme oxygenase-1. FEBS Open Bio.

[121] Yoshinaga T., Sassa S., Kappas A. (1982). The oxidative degradation of heme c by the microsomal heme oxygenase system. J. Biol. Chem..

[122] Chiabrando D., Vinchi F., Fiorito V., Mercurio S., Tolosano E. (2014). Heme in pathophysiology: A matter of scavenging, metabolism and trafficking across cell membranes. Front. Pharmacol..

[123] Soares M.P., Bozza M.T. (2016). Red alert: Labile heme is an alarmin. Curr. Opin. Immunol..

[124] Tudor C., Lerner-Marmarosh N., Engelborghs Y., Gibbs P.E., Maines M.D. (2008). Biliverdin reductase is a transporter of haem into the nucleus and is essential for regulation of HO-1 gene expression by haematin. Biochem. J..

[125] Vincent S.H., Muller-Eberhard U. (1985). A protein of the Z class of liver cytosolic proteins in the rat that preferentially binds heme. J. Biol. Chem..

[126] Taketani S., Adachi Y., Kohno H., Ikehara S., Tokunaga R., Ishii T. (1998). Molecular characterization of a newly identified heme-binding protein induced during differentiation of urine erythroleukemia cells. J. Biol. Chem..

[127] Smith A., McCulloh R.J. (2015). Hemopexin and haptoglobin: Allies against heme toxicity from hemoglobin not contenders. Front. Physiol..

[128] Gutteridge J.M., Smith A. (1988). Antioxidant protection by haemopexin of haem-stimulated lipid peroxidation. Biochem. J..

[129] Balla G., Jacob H.S., Eaton J.W., Belcher J.D., Vercellotti G.M. (1991). Hemin: A possible physiological mediator of low density lipoprotein oxidation and endothelial injury. Arterioscler. Thromb..

[130] Jeney V., Balla J., Yachie A., Varga Z., Vercellotti G.M., Eaton J.W., Balla G. (2002). Pro-oxidant and cytotoxic effects of circulating heme. Blood.

[131] Nagy E., Eaton J.W., Jeney V., Soares M.P., Varga Z., Galajda Z., Szentmiklosi J., Mehes G., Csonka T., Smith A. (2010). Red cells, hemoglobin, heme, iron, and atherogenesis. Arterioscler. Thromb. Vasc. Biol..

[132] Simoes R.L., Arruda M.A., Canetti C., Serezani C.H., Fierro I.M., Barja-Fidalgo C. (2013). Proinflammatory responses of heme in alveolar macrophages: Repercussion in lung hemorrhagic episodes. Mediators. Inflamm..

[133] Fernandez P.L., Dutra F.F., Alves L., Figueiredo R.T., Mourao-Sa D., Fortes G.B., Bergstrand S., Lonn D., Cevallos R.R., Pereira R.M. (2010). Heme amplifies the innate immune response to microbial molecules through spleen tyrosine kinase (Syk)-dependent reactive oxygen species generation. J. Biol. Chem..

[134] Dutra F.F., Alves L.S., Rodrigues D., Fernandez P.L., de Oliveira R.B., Golenbock D.T., Zamboni D.S., Bozza M.T. (2014). Hemolysis-induced lethality involves inflammasome activation by heme. Proc. Natl. Acad. Sci. USA.

[135] Fortes G.B., Alves L.S., de Oliveira R., Dutra F.F., Rodrigues D., Fernandez P.L., Souto-Padron T., De Rosa M.J., Kelliher M., Golenbock D. (2012). Heme induces programmed necrosis on macrophages through autocrine TNF and ROS production. Blood.

[136] Vinchi F., Costa da S.M., Ingoglia G., Petrillo S., Brinkman N., Zuercher A., Cerwenka A., Tolosano E., Muckenthaler M.U. (2016). Hemopexin therapy reverts heme-induced proinflammatory phenotypic switching of macrophages in a mouse model of sickle cell disease. Blood.

[137] Wegiel B., Hauser C.J., Otterbein L.E. (2015). Heme as a danger molecule in pathogen recognition. Free Radic. Biol. Med..

[138] Martins R., Maier J., Gorki A.D., Huber K.V., Sharif O., Starkl P., Saluzzo S., Quattrone F., Gawish R., Lakovits K. (2016). Heme drives hemolysis-induced susceptibility to infection via disruption of phagocyte functions. Nat. Immunol..

[139] Doty R.T., Phelps S.R., Shadle C., Sanchez-Bonilla M., Keel S.B., Abkowitz J.L. (2015). Coordinate expression of heme and globin is essential for effective erythropoiesis. J. Clin. Investig..

[140] Klouche K., Morena M., Canaud B., Descomps B., Beraud J.J., Cristol J.P. (2004). Mechanism of in vitro heme-induced LDL oxidation: Effects of antioxidants. Eur. J. Clin. Investig..

[141] Schaer D.J., Buehler P.W., Alayash A.I., Belcher J.D., Vercellotti G.M. (2013). Hemolysis and free hemoglobin revisited: Exploring hemoglobin and hemin scavengers as a novel class of therapeutic proteins. Blood.

[142] Hirschhorn T., Stockwell B.R. (2019). The development of the concept of ferroptosis. Free Radic. Biol. Med..

[143] Toppo S., Flohe L., Ursini F., Vanin S., Maiorino M. (2009). Catalytic mechanisms and specificities of glutathione peroxidases: Variations of a basic scheme. Biochim. Biophys. Acta.

[144] Pizzimenti S., Ciamporcero E., Daga M., Pettazzoni P., Arcaro A., Cetrangolo G., Minelli R., Dianzani C., Lepore A., Gentile F. (2013). Interaction of aldehydes derived from lipid peroxidation and membrane proteins. Front. Physiol..

[145] Dungel P., Perlinger M., Weidinger A., Redl H., Kozlov A.V. (2015). The cytoprotective effect of nitrite is based on the formation of dinitrosyl iron complexes. Free Radic. Biol. Med..

[146] Fotiou S., Fotiou D., Deliconstantinos G. (2009). Formation of heme-iron complexes with nitric oxide (NO) and peroxynitrite (ONOO-) after ultraviolet radiation as a protective mechanism in rat skin. In Vivo.

[147] Sahni S., Hickok J.R., Thomas D.D. (2018). Nitric oxide reduces oxidative stress in cancer cells by forming dinitrosyliron complexes. Nitric Oxide.

[148] Deliconstantinos G., Villiotou V., Stavrides J.C. (1994). Scavenging effects of hemoglobin and related heme containing compounds on nitric oxide, reactive oxidants and carcinogenic volatile nitrosocompounds of cigarette smoke. A new method for protection against the dangerous cigarette constituents. Anticancer Res..

[149] Willis D., Tomlinson A., Frederick R., Paul-Clark M.J., Willoughby D.A. (1995). Modulation of heme oxygenase activity in rat brain and spleen by inhibitors and donors of nitric oxide. Biochem. Biophys. Res. Commun..

[150] Murphy M.P. (1999). Nitric oxide and cell death. Biochim. Biophys. Acta.

[151] Rus A., Molina F., Peinado M.A., Del Moral M.L. (2010). Endogenous nitric oxide can act as beneficial or deleterious in the hypoxic lung depending on the reoxygenation time. Anat. Rec..

[152] van Faassen E.E., Bahrami S., Feelisch M., Hogg N., Kelm M., Kim-Shapiro D.B., Kozlov A.V., Li H., Lundberg J.O., Mason R. (2009). Nitrite as regulator of hypoxic signaling in mammalian physiology. Med. Res. Rev..

[153] Lundberg J.O., Gladwin M.T., Ahluwalia A., Benjamin N., Bryan N.S., Butler A., Cabrales P., Fago A., Feelisch M., Ford P.C. (2009). Nitrate and nitrite in biology, nutrition and therapeutics1. Nat. Chem. Biol..

[154] Kitamuro T., Takahashi K., Ogawa K., Udono-Fujimori R., Takeda K., Furuyama K., Nakayama M., Sun J., Fujita H., Hida W. (2003). Bach1 functions as a hypoxia-inducible repressor for the heme oxygenase-1 gene in human cells. J. Biol. Chem..

[155] Ishikawa M., Numazawa S., Yoshida T. (2005). Redox regulation of the transcriptional repressor Bach1. Free Radic. Biol. Med..

[156] Igarashi K., Itoh K., Hayashi N., Nishizawa M., Yamamoto M. (1995). Conditional expression of the ubiquitous transcription factor MafK induces erythroleukemia cell differentiation. Proc. Natl. Acad. Sci. USA.

[157] Ishii T., Itoh K., Takahashi S., Sato H., Yanagawa T., Katoh Y., Bannai S., Yamamoto M. (2000). Transcription factor Nrf2 coordinately regulates a group of oxidative stress-inducible genes in macrophages. J. Biol. Chem..

[158] Itoh K., Chiba T., Takahashi S., Ishii T., Igarashi K., Katoh Y., Oyake T., Hayashi N., Satoh K., Hatayama I. (1997). An Nrf2/small Maf heterodimer mediates the induction of phase II detoxifying enzyme genes through antioxidant response elements. Biochem. Biophys. Res. Commun..

[159] Tahara T., Sun J., Nakanishi K., Yamamoto M., Mori H., Saito T., Fujita H., Igarashi K., Taketani S. (2004). Heme positively regulates the expression of beta-globin at the locus control region via the transcriptional factor Bach1 in erythroid cells. J. Biol. Chem..

[160] Warnatz H.J., Schmidt D., Manke T., Piccini I., Sultan M., Borodina T., Balzereit D., Wruck W., Soldatov A., Vingron M. (2011). The BTB and CNC homology 1 (BACH1) target genes are involved in the oxidative stress response and in control of the cell cycle. J. Biol. Chem..

[161] Hira S., Tomita T., Matsui T., Igarashi K., Ikeda-Saito M. (2007). Bach1, a heme-dependent transcription factor, reveals presence of multiple heme binding sites with distinct coordination structure. IUBMB Life.

[162] Ogawa K., Sun J., Taketani S., Nakajima O., Nishitani C., Sassa S., Hayashi N., Yamamoto M., Shibahara S., Fujita H. (2001). Heme mediates derepression of Maf recognition element through direct binding to transcription repressor Bach1. Embo J..

[163] Shimizu T. (2012). Binding of cysteine thiolate to the Fe(III) heme complex is critical for the function of heme sensor proteins. J. Inorg. Biochem..

[164] Wissbrock A., Paul George A.A., Brewitz H.H., Kuhl T., Imhof D. (2019). The molecular basis of transient heme-protein interactions: Analysis, concept and implementation. Biosci. Rep..

[165] Zenke-Kawasaki Y., Dohi Y., Katoh Y., Ikura T., Ikura M., Asahara T., Tokunaga F., Iwai K., Igarashi K. (2007). Heme induces ubiquitination and degradation of the transcription factor Bach1. Mol. Cell Biol..

[166] Yamasaki C., Tashiro S., Nishito Y., Sueda T., Igarashi K. (2005). Dynamic cytoplasmic anchoring of the transcription factor Bach1 by intracellular hyaluronic acid binding protein IHABP. J. Biochem..

[167] Igarashi K., Kataoka K., Itoh K., Hayashi N., Nishizawa M., Yamamoto M. (1994). Regulation of transcription by dimerization of erythroid factor NF-E2 p45 with small Maf proteins. Nature.

[168] Itoh K., Wakabayashi N., Katoh Y., Ishii T., O’Connor T., Yamamoto M. (2003). Keap1 regulates both cytoplasmic-nuclear shuttling and degradation of Nrf2 in response to electrophiles. Genes Cells.

[169] Marro S., Chiabrando D., Messana E., Stolte J., Turco E., Tolosano E., Muckenthaler M.U. (2010). Heme controls ferroportin1 (FPN1) transcription involving Bach1, Nrf2 and a MARE/ARE sequence motif at position -7007 of the FPN1 promoter. Haematologica.

[170] Nandakumar S.K., Ulirsch J.C., Sankaran V.G. (2016). Advances in understanding erythropoiesis: Evolving perspectives. Br. J. Haematol..

[171] Valent P., Busche G., Theurl I., Uras I.Z., Germing U., Stauder R., Sotlar K., Fureder W., Bettelheim P., Pfeilstocker M. (2018). Normal and pathological erythropoiesis in adults: From gene regulation to targeted treatment concepts. Haematologica.

[172] Palis J. (2014). Primitive and definitive erythropoiesis in mammals. Front. Physiol..

[173] Koury M.J., Bondurant M.C. (1990). Erythropoietin retards DNA breakdown and prevents programmed death in erythroid progenitor cells. Science.

[174] Evans T., Felsenfeld G. (1989). The erythroid-specific transcription factor Eryf1: A new finger protein. Cell.

[175] Sadlon T.J., Dell’Oso T., Surinya K.H., May B.K. (1999). Regulation of erythroid 5-aminolevulinate synthase expression during erythropoiesis. Int. J. Biochem. Cell Biol..

[176] Tanimura N., Miller E., Igarashi K., Yang D., Burstyn J.N., Dewey C.N., Bresnick E.H. (2016). Mechanism governing heme synthesis reveals a GATA factor/heme circuit that controls differentiation. Embo Rep..

[177] Keel S.B., Doty R.T., Yang Z., Quigley J.G., Chen J., Knoblaugh S., Kingsley P.D., De D.I., Vaughn M.B., Kaplan J. (2008). A heme export protein is required for red blood cell differentiation and iron homeostasis. Science.

[178] Quigley J.G., Yang Z., Worthington M.T., Phillips J.D., Sabo K.M., Sabath D.E., Berg C.L., Sassa S., Wood B.L., Abkowitz J.L. (2004). Identification of a human heme exporter that is essential for erythropoiesis. Cell.

[179] Mercurio S., Petrillo S., Chiabrando D., Bassi Z.I., Gays D., Camporeale A., Vacaru A., Miniscalco B., Valperga G., Silengo L. (2015). The heme exporter Flvcr1 regulates expansion and differentiation of committed erythroid progenitors by controlling intracellular heme accumulation. Haematologica.

[180] Khan A.A., Quigley J.G. (2013). Heme and FLVCR-related transporter families SLC48 and SLC49. Mol. Asp. Med..

[181] Kobayashi M., Kato H., Hada H., Itoh-Nakadai A., Fujiwara T., Muto A., Inoguchi Y., Ichiyanagi K., Hojo W., Tomosugi N. (2017). Iron-heme-Bach1 axis is involved in erythroblast adaptation to iron deficiency. Haematologica.

[182] Han A.P., Yu C., Lu L., Fujiwara Y., Browne C., Chin G., Fleming M., Leboulch P., Orkin S.H., Chen J.J. (2001). Heme-regulated eIF2alpha kinase (HRI) is required for translational regulation and survival of erythroid precursors in iron deficiency. Embo J..

[183] Taniuchi S., Miyake M., Tsugawa K., Oyadomari M., Oyadomari S. (2016). Integrated stress response of vertebrates is regulated by four eIF2alpha kinases. Sci. Rep..

[184] Suragani R.N., Zachariah R.S., Velazquez J.G., Liu S., Sun C.W., Townes T.M., Chen J.J. (2012). Heme-regulated eIF2alpha kinase activated Atf4 signaling pathway in oxidative stress and erythropoiesis. Blood.

[185] Zhang S., Macias-Garcia A., Ulirsch J.C., Velazquez J., Butty V.L., Levine S.S., Sankaran V.G., Chen J.J. (2019). HRI coordinates translation necessary for protein homeostasis and mitochondrial function in erythropoiesis. Elife.

[186] Yin L., Wu N., Curtin J.C., Qatanani M., Szwergold N.R., Reid R.A., Waitt G.M., Parks D.J., Pearce K.H., Wisely G.B. (2007). Rev-erbalpha, a heme sensor that coordinates metabolic and circadian pathways. Science.

[187] Li B., Takeda K., Ishikawa K., Yoshizawa M., Sato M., Shibahara S., Furuyama K. (2012). Coordinated expression of 6-phosphofructo-2-kinase/fructose-2,6-bisphosphatase 4 and heme oxygenase 2: Evidence for a regulatory link between glycolysis and heme catabolism. Tohoku J. Exp. Med..

[188] Chesney J., Clark J., Klarer A.C., Imbert-Fernandez Y., Lane A.N., Telang S. (2014). Fructose-2,6-bisphosphate synthesis by 6-phosphofructo-2-kinase/fructose-2,6-bisphosphatase 4 (PFKFB4) is required for the glycolytic response to hypoxia and tumor growth 1. Oncotarget.

[189] Padmanaban G., Venkateswar V., Rangarajan P.N. (1989). Haem as a multifunctional regulator. Trends Biochem. Sci..

[190] Atamna H. (2004). Heme, iron, and the mitochondrial decay of ageing. Ageing Res. Rev..

[191] Kimpara T., Takeda A., Yamaguchi T., Arai H., Okita N., Takase S., Sasaki H., Itoyama Y. (2000). Increased bilirubins and their derivatives in cerebrospinal fluid in Alzheimer’s disease. Neurobiol. Aging.

[192] Atamna H., Liu J., Ames B.N. (2001). Heme deficiency selectively interrupts assembly of mitochondrial complex IV in human fibroblasts: Revelance to aging. J. Biol. Chem..

[193] Rogers P.M., Ying L., Burris T.P. (2008). Relationship between circadian oscillations of Rev-erbalpha expression and intracellular levels of its ligand, heme. Biochem. Biophys. Res. Commun..

[194] Bailey S.M., Udoh U.S., Young M.E. (2014). Circadian regulation of metabolism. J. Endocrinol..

[195] Takahashi J.S. (2015). Molecular components of the circadian clock in mammals. Diabetes Obes. Metab..

[196] Raghuram S., Stayrook K.R., Huang P., Rogers P.M., Nosie A.K., McClure D.B., Burris L.L., Khorasanizadeh S., Burris T.P., Rastinejad F. (2007). Identification of heme as the ligand for the orphan nuclear receptors REV-ERBalpha and REV-ERBbeta. Nat. Struct. Mol. Biol..

[197] Wu N., Yin L., Hanniman E.A., Joshi S., Lazar M.A. (2009). Negative feedback maintenance of heme homeostasis by its receptor, Rev-erbalpha. Genes Dev..

[198] Scrima R., Cela O., Merla G., Augello B., Rubino R., Quarato G., Fugetto S., Menga M., Fuhr L., Relogio A. (2016). Clock-genes and mitochondrial respiratory activity: Evidence of a reciprocal interplay. Biochim. Biophys. Acta.

[199] Cela O., Scrima R., Pazienza V., Merla G., Benegiamo G., Augello B., Fugetto S., Menga M., Rubino R., Fuhr L. (2016). Clock genes-dependent acetylation of complex I sets rhythmic activity of mitochondrial OxPhos. Biochim. Biophys. Acta.

[200] Mullebner A., Dorighello G.G., Kozlov A.V., Duvigneau J.C. (2017). Interaction between Mitochondrial Reactive Oxygen Species, Heme Oxygenase, and Nitric Oxide Synthase Stimulates Phagocytosis in Macrophages. Front. Med. (Lausanne).

[201] Zhang T., Bu P., Zeng J., Vancura A. (2017). Increased heme synthesis in yeast induces a metabolic switch from fermentation to respiration even under conditions of glucose repression. J. Biol. Chem..

[202] Lin P.H., Chiang M.T., Chau L.Y. (2008). Ubiquitin-proteasome system mediates heme oxygenase-1 degradation through endoplasmic reticulum-associated degradation pathway. Biochim. Biophys. Acta.

[203] Broca C., Varin E., Armanet M., Tourrel-Cuzin C., Bosco D., Dalle S., Wojtusciszyn A. (2014). Proteasome dysfunction mediates high glucose-induced apoptosis in rodent beta cells and human islets. PLoS ONE.

[204] Dong C., Zheng H., Huang S., You N., Xu J., Ye X., Zhu Q., Feng Y., You Q., Miao H. (2015). Heme oxygenase-1 enhances autophagy in podocytes as a protective mechanism against high glucose-induced apoptosis. Exp. Cell Res..

[205] Almeida A.S., Figueiredo-Pereira C., Vieira H.L. (2015). Carbon monoxide and mitochondria-modulation of cell metabolism, redox response and cell death. Front. Physiol..

[206] Kaczara P., Motterlini R., Rosen G.M., Augustynek B., Bednarczyk P., Szewczyk A., Foresti R., Chlopicki S. (2015). Carbon monoxide released by CORM-401 uncouples mitochondrial respiration and inhibits glycolysis in endothelial cells: A role for mitoBKCa channels. Biochim. Biophys. Acta.

[207] Braud L., Pini M., Muchova L., Manin S., Kitagishi H., Sawaki D., Czibik G., Ternacle J., Derumeaux G., Foresti R. (2018). Carbon monoxide-induced metabolic switch in adipocytes improves insulin resistance in obese mice. JCI Insight.

[208] Yoshida T., Kikuchi G. (1978). Purification and properties of heme oxygenase from pig spleen microsomes. J. Biol. Chem..

[209] Wagner G., Lindroos-Christensen J., Einwallner E., Husa J., Zapf T.C., Lipp K., Rauscher S., Groger M., Spittler A., Loewe R. (2017). HO-1 inhibits preadipocyte proliferation and differentiation at the onset of obesity via ROS dependent activation of Akt2. Sci. Rep..

[210] Gordon S., Pluddemann A. (2017). Tissue macrophages: Heterogeneity and functions. BMC Biol..

[211] Davies L.C., Jenkins S.J., Allen J.E., Taylor P.R. (2013). Tissue-resident macrophages. Nat. Immunol..

[212] Gordon S., Taylor P.R. (2005). Monocyte and macrophage heterogeneity. Nat. Rev. Immunol..

[213] Naito Y., Takagi T., Higashimura Y. (2014). Heme oxygenase-1 and anti-inflammatory M2 macrophages. Arch. Biochem. Biophys..

[214] Immenschuh S., Vijayan V., Janciauskiene S., Gueler F. (2017). Heme as a Target for Therapeutic Interventions. Front. Pharmacol..

[215] Hull T.D., Agarwal A., George J.F. (2014). The mononuclear phagocyte system in homeostasis and disease: A role for heme oxygenase-1. Antioxid. Redox. Signal..

[216] Lee T.S., Chau L.Y. (2002). Heme oxygenase-1 mediates the anti-inflammatory effect of interleukin-10 in mice. Nat. Med..

[217] Kartikasari A.E., Wagener F.A., Yachie A., Wiegerinck E.T., Kemna E.H., Swinkels D.W. (2009). Hepcidin suppression and defective iron recycling account for dysregulation of iron homeostasis in heme oxygenase-1 deficiency. J. Cell Mol. Med..

[218] Puri N., Arefiev Y., Chao R., Sacerdoti D., Chaudry H., Nichols A., Srikanthan K., Nawab A., Sharma D., Lakhani V.H. (2017). Heme Oxygenase Induction Suppresses Hepatic Hepcidin and Rescues Ferroportin and Ferritin Expression in Obese Mice. J. Nutr. Metab..

[219] Philip M., Chiu E.Y., Hajjar A.M., Abkowitz J.L. (2016). TLR Stimulation Dynamically Regulates Heme and Iron Export Gene Expression in Macrophages. J. Immunol. Res..

[220] Khan A.A., Quigley J.G. (2011). Control of intracellular heme levels: Heme transporters and heme oxygenases. Biochim. Biophys. Acta.

[221] Kortman G.A., Mulder M.L., Richters T.J., Shanmugam N.K., Trebicka E., Boekhorst J., Timmerman H.M., Roelofs R., Wiegerinck E.T., Laarakkers C.M. (2015). Low dietary iron intake restrains the intestinal inflammatory response and pathology of enteric infection by food-borne bacterial pathogens. Eur. J. Immunol..

[222] Zhang X., Mosser D.M. (2008). Macrophage activation by endogenous danger signals. J. Pathol..

[223] Oppenheim J.J., Yang D. (2005). Alarmins: Chemotactic activators of immune responses. Curr. Opin. Immunol..

[224] Bianchi M.E. (2007). DAMPs, PAMPs and alarmins: All we need to know about danger. J. Leukoc. Biol..

[225] Flannagan R.S., Jaumouille V., Grinstein S. (2012). The cell biology of phagocytosis. Annu. Rev. Pathol..

[226] Lin Q., Weis S., Yang G., Weng Y.H., Helston R., Rish K., Smith A., Bordner J., Polte T., Gaunitz F. (2007). Heme oxygenase-1 protein localizes to the nucleus and activates transcription factors important in oxidative stress. J. Biol. Chem..

[227] McCoubrey W.K., Eke B., Maines M.D. (1995). Multiple transcripts encoding heme oxygenase-2 in rat testis: Developmental and cell-specific regulation of transcripts and protein. Biol. Reprod..

[228] McCoubrey W.K., Huang T.J., Maines M.D. (1997). Heme oxygenase-2 is a hemoprotein and binds heme through heme regulatory motifs that are not involved in heme catalysis. J. Biol. Chem..

[229] Ragsdale S.W., Yi L. (2011). Thiol/Disulfide redox switches in the regulation of heme binding to proteins. Antioxid. Redox. Signal..

[230] Yi L., Jenkins P.M., Leichert L.I., Jakob U., Martens J.R., Ragsdale S.W. (2009). Heme regulatory motifs in heme oxygenase-2 form a thiol/disulfide redox switch that responds to the cellular redox state. J. Biol. Chem..

[231] Boehning D., Moon C., Sharma S., Hurt K.J., Hester L.D., Ronnett G.V., Shugar D., Snyder S.H. (2003). Carbon monoxide neurotransmission activated by CK2 phosphorylation of heme oxygenase-2. Neuron.

[232] Boehning D., Sedaghat L., Sedlak T.W., Snyder S.H. (2004). Heme oxygenase-2 is activated by calcium-calmodulin. J. Biol. Chem..

[233] Weng Y.H., Yang G., Weiss S., Dennery P.A. (2003). Interaction between heme oxygenase-1 and -2 proteins. J. Biol. Chem..

[234] Barone E., Di D.F., Sultana R., Coccia R., Mancuso C., Perluigi M., Butterfield D.A. (2012). Heme oxygenase-1 posttranslational modifications in the brain of subjects with Alzheimer disease and mild cognitive impairment. Free Radic. Biol. Med..

[235] Kinobe R., Ji Y., Nakatsu K. (2004). Peroxynitrite-mediated inactivation of heme oxygenases. BMC Pharmacol..

[236] Sardana M.K., Kappas A. (1987). Dual control mechanism for heme oxygenase: Tin(IV)-protoporphyrin potently inhibits enzyme activity while markedly increasing content of enzyme protein in liver. Proc. Natl. Acad. Sci. USA.

[237] El-Brolosy M.A., Stainier D.Y.R. (2017). Genetic compensation: A phenomenon in search of mechanisms. PLoS Genet..

[238] Chang E.F., Wong R.J., Vreman H.J., Igarashi T., Galo E., Sharp F.R., Stevenson D.K., Noble-Haeusslein L.J. (2003). Heme oxygenase-2 protects against lipid peroxidation-mediated cell loss and impaired motor recovery after traumatic brain injury. J. Neurosci..

